# Entorhinal Astrocyte Transplants Restore Spatial Exploration and Alleviate Amyloid‐Beta Pathology in Alzheimer's Mice

**DOI:** 10.1002/advs.77139

**Published:** 2026-08-24

**Authors:** Fengjuan Wu, Jihua Fan, Xiang Zhou, Zhiqi Yang, Mingyue Gong, Mingzhu Huang, Zhenlu Cai, Shaofan Yang, Teng Teng, Chuanyan Yang, Jin Li, Haoyu Wang, Shuangshuang Dai, Chunhai Chen, Xiaowei Chen, Manxia Wang, Kuan Zhang

**Affiliations:** ^1^ Brain Research Center and State Key Laboratory of Trauma and Chemical Poisoning Third Military Medical University Chongqing China; ^2^ Department of Neurology Lanzhou University Second Hospital Lanzhou China; ^3^ Guangxi Key Laboratory of Special Biomedicine/Advanced Institute for Brain and Intelligence School of Medicine Guangxi University Nanning China; ^4^ Department of Neurology Gansu Provincial Central Hospital Lanzhou China; ^5^ College of Bioengineering Chongqing University Chongqing China; ^6^ Department of Neurosurgery Southwest Hospital Third Military Medical University Chongqing China; ^7^ Department of Biochemistry and Molecular Biology School of Basic Medicine Army Medical University Chongqing China; ^8^ Department of Occupational Health Third Military Medical University Chongqing China

**Keywords:** Alzheimer’s disease, AQP4, astrocyte, glial progenitor cell, medial entorhinal cortex, neuroinflammation, spatial exploration

## Abstract

Astrocytes in the medial entorhinal cortex (MEC) regulate spatial exploration, yet their functional impairment in Alzheimer's disease (AD) remains poorly understood. Here, we show that fragmented spatial exploration in APP/PS1 mice correlates with diminished exploration‐evoked Ca^2+^ transients in MEC layer II astrocytes. To address this, we transplanted glial progenitor cells into the MEC of aged AD mice. The engrafted cells differentiated into homeostatic astrocytes and restored perivascular Aquaporin‐4 polarization. This remodeling significantly reduced amyloid‐beta burden, attenuated neuroinflammation, and preserved synaptic integrity. Crucially, these structural and molecular improvements specifically reversed spatial exploration deficits without affecting general locomotion. Together, our findings suggest that dysfunction in MECII astrocytes is associated with fragmented exploratory behavior. Furthermore, the region‐specific replacement of astrocytes may ameliorate the spatial exploration deficits observed in the AD mouse model.

## Graphical abstract

Impaired MEC astrocytic Ca^2^^+^ signaling is associated with fragmented spatial exploration in AD mice. Region‐specific glial progenitor transplantation generates engrafted astrocytes that are accompanied by improved AQP4 polarization, reduced amyloid‐β‐associated pathology, attenuated neuroinflammation, preserved synaptic integrity, and ameliorated spatial exploration deficits, supporting a proof‐of‐concept astrocyte‐replacement strategy.

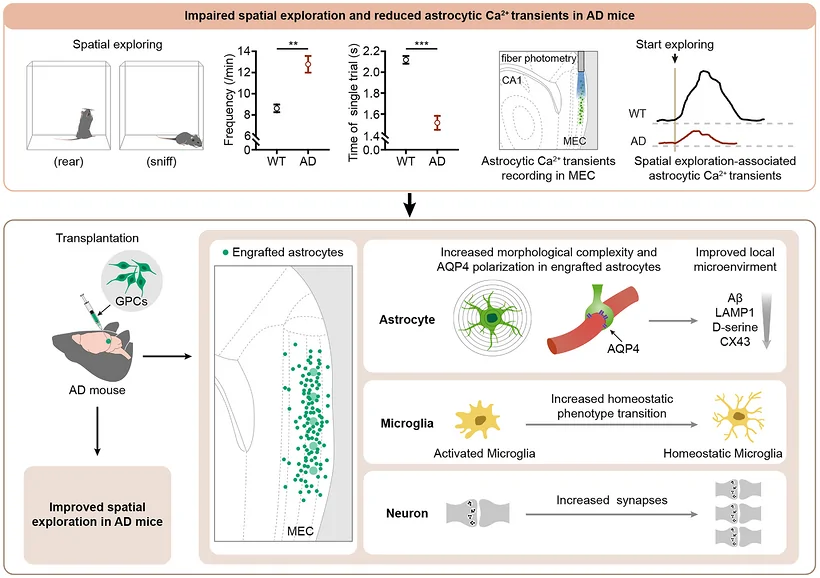

**Abbreviations**
AAV: Adeno‐Associated Virus
Aβ: Amyloid‐beta
AD: Alzheimer's disease
ALS: Amyotrophic Lateral Sclerosis
AQP4: Aquaporin‐4
CNS: Central nervous system
CNTF: Ciliary neurotrophic factor
CX43: Connexin 43
Cxs: Connexins
DAPI: 4′,6‐diamidino‐2‐phenylindole
EGCs: Engrafted enteric glial cells
EGF: Epidermal growth factor
FBS: Fetal bovine serum
FGF2: Fibroblast growth factor 2
GECIs: Genetically encoded calcium indicators
GPCs: Glial progenitor cells
GVU: Glial‐vascular unit
HD: Huntington's Disease
LAMP1: Lysosome‐associated membrane protein 1
MEC: Medial entorhinal cortex
MECII: Medial entorhinal cortex layer II
NSCs: Neural stem cells
OFT: Open Field Test
PBS: Phosphate‐buffered saline
PCA: Principal component analysis
PD: Parkinson's Disease
PFA: Paraformaldehyde
SEM: Standard Error of the Mean
SYP: Synaptophysin
TBI: Traumatic brain injury
WT: Wild‐type
ZEN: Zeiss Efficient Navigation

## 1 Introduction

Spatial exploration behaviors, such as sniffing and rearing, are essential for gathering and processing environmental information [1]. These actions are driven by intrinsic motivations, arousal levels, and fear responses [2]. They play a critical role in survival by enhancing awareness of the environment, aiding in food detection, and helping to avoid danger [3]. Additionally, spatial exploration constitutes a significant form of cognitive behavior that supports learning processes [3] and serves as the foundation for learning and memory [3]. Therefore, spatial exploration is not only vital for survival and adaptation to the environment but also crucial for learning and memory. Research indicates that Alzheimer's disease (AD) impairs spatial exploratory behaviors [4, 5, 6, 7] and diminishes responses to novel environments [5]. Consequently, deficits in spatial exploration represent a hallmark behavioral symptom of AD.

Astrocytes constitute a major cell population throughout the nervous system [8]. Each astrocyte interacts with a large number of synapses—approximately 90 000 in rodents and 2 million in humans [9]. This vast network positions astrocytes as central hubs for integrating information from multiple sources, allowing them to modulate neuronal activity across both spatial and temporal dimensions [10, 11]. Huang et al. demonstrated that spatial exploration activates astrocytes in the medial entorhinal cortex (MEC). These astrocytic activities, induced by spatial exploration, enhance spatial exploratory behaviors and support the functioning of MEC neurons [12]. This finding underscores the critical role of MEC astrocytes in spatial exploration. The MEC itself is particularly vulnerable in AD [13, 14]. In AD‐affected brains, astrocytes undergo both atrophic and reactive changes, exhibiting Ca^2^^+^ hyperactivity in a region‐dependent manner [15, 16]. In the MEC, cognitive decline in AD correlates with a marked reduction in astrocytic branching and a contraction of the astrocyte domain [17, 18]. Despite these changes, it remains unclear whether astrocytic activities linked to spatial exploration are disrupted in the MEC of AD animals that display impaired spatial exploratory behaviors.

Brain disorders often involve significant glial pathologies. Cell transplantation has emerged as a promising therapeutic strategy for a variety of neurological conditions, offering the potential to restore lost functions and regenerate brain tissue [19]. Recently, astrocyte transplantation has gained attention as a viable treatment option [20]. By replacing apoptotic astrocytes, this approach may reduce inflammation, slow disease progression, and improve functional outcomes in translational models of disorders such as Amyotrophic Lateral Sclerosis (ALS) [21, 22], Parkinson's Disease (PD) [23, 24], AD [25, 26], Huntington's Disease (HD) [27], ischemic events [28], and traumatic brain injury (TBI) [29, 30]. These findings support the potential of astrocyte transplantation as an effective intervention for numerous brain disorders.

Reactive astrogliosis, characterized by morphological and molecular changes in astrocytes, often precedes neurodegeneration, suggesting its role in the progression of AD. This highlights the potential of targeting astrocytes as a therapeutic strategy for AD [31]. Previous studies have demonstrated that transplanting human glial progenitor cells (GPCs) enhances long‐term potentiation and improves cognitive function [32]. The engrafted astrocytes not only integrated long‐term but also countered age‐related declines in sensory responses [33]. Furthermore, these astrocytes were able to internalize human amyloid‐beta (Aβ) immunoreactive material in vivo [25]. Similarly, engrafted enteric glial cells (EGCs) exhibited anti‐amyloidogenic properties, reducing Aβ‐induced neuroinflammation and neurodegeneration [26]. Additional research in a mouse model of human tauopathy further supports the neuroprotective role of engrafted astrocytes [34]. However, it remains unclear whether astrocyte transplantation can ameliorate the spatial exploration deficits associated with AD.

The aim of this study was to examine the functional changes in MEC astrocytes during spatial exploration and evaluate the effects of engrafted astrocytes on both the pathological environment and spatial exploratory behaviors in AD mice. Using cell‐specific genetically encoded calcium indicators (GECIs) combined with fiber photometry [35, 36, 37], we monitored astrocytic activity in the MEC of AD mice during free spatial exploration. We also transplanted GPCs into the MEC to assess the integration of engrafted astrocytes and their impact on the pathological environment and spatial exploration deficits in AD mice. Our results demonstrate reduced astrocytic activity within MECII in AD mice, which is associated with fragmented spatial exploration. The long‐term survival of transplanted astrocytes correlated with enhanced perivascular AQP4 polarization, coinciding with a lower Aβ burden, reduced local neuroinflammation, preserved synaptic integrity, and improved spatial exploratory behaviors. Our findings offer new insights into the potential of astrocyte transplantation as a therapeutic strategy to mitigate the progression of AD.

## 2 Results

### 2.1 Fragmented Spatial Exploration in AD Mice Is Associated With Reduced Astrocytic Ca^2+^ Transients in the Medial Entorhinal Cortex Layer II (MECII)

**FIGURE 1:**
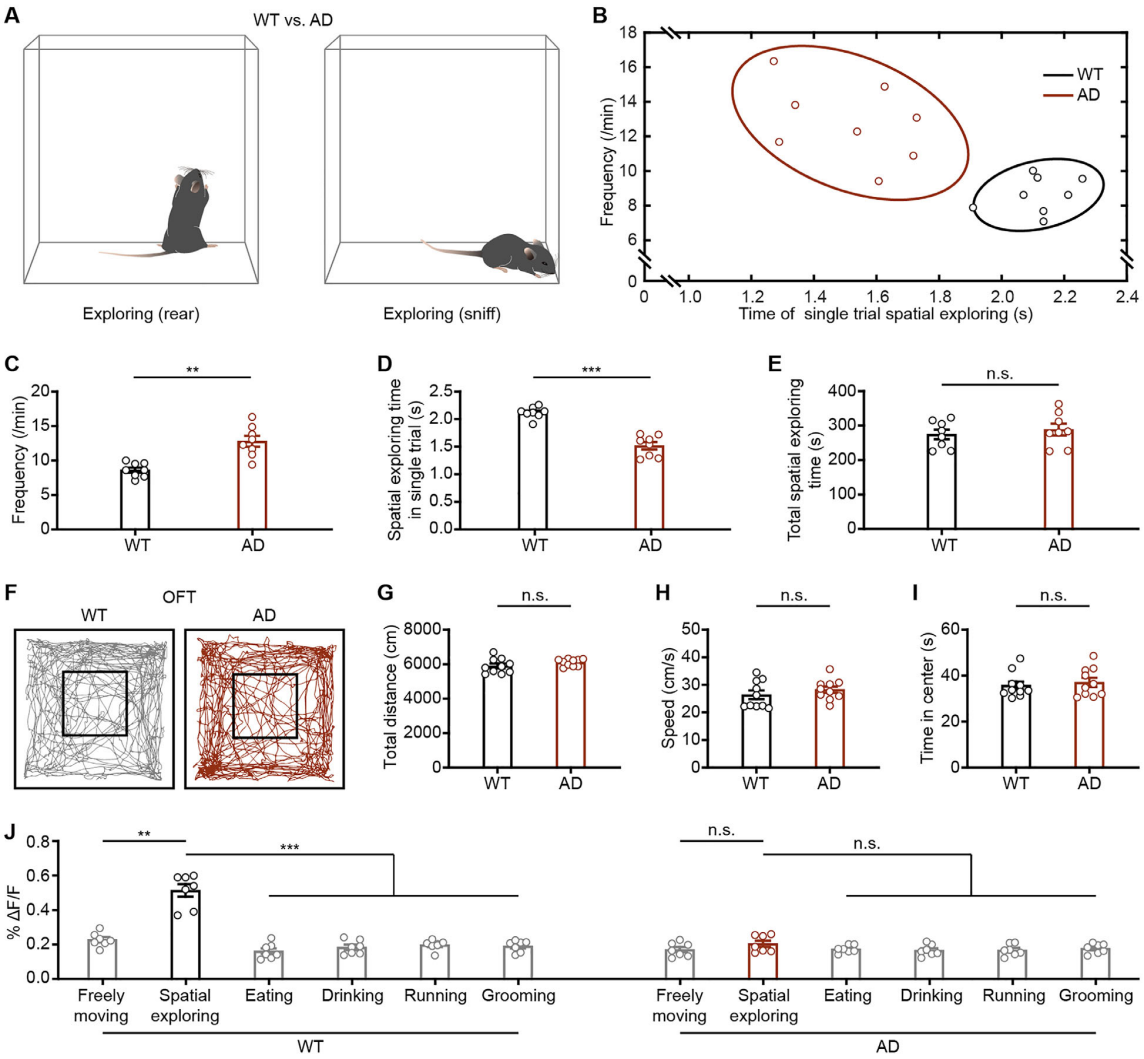
Fragmented spatial exploration in AD mice is associated with altered behavior‐specific MECII astrocytic Ca^2^^+^ transients. (A) Schematic illustrating analyzed exploratory behaviors of WT and AD mice, including rearing (left) and sniffing (right). (B) Principal component analysis between spatial exploration time and frequency in WT and AD mice (n = 8 mice per group). (C–E) Histograms summarizing frequency (C), spatial exploration time in a single trial (D), and total spatial exploration time (E) of WT and AD mice (*n* = 8 mice per group). (F) Representative trajectory plots of OFT in WT and AD mice. Black box represents the central region. (G–I) Histograms summarizing total distance (G), locomotion speed (H), and time spent in the central area (I) during OFT in WT and AD mice (*n* = 10 mice per group). Independent samples via the Wilcoxon rank sum test. (J) Histogram showing amplitudes of the MECII astrocyte Ca^2^^+^ transients in WT and AD mice during distinct, spontaneously occurring behaviors. Different behavioral situations are covered, including free movement, spatial exploration, eating, drinking, running, and grooming (*n* = 7 mice per group); *p < 0.05, **p < 0.01, and ***p < 0.001, via one‐way ANOVA with Tamhane's T2 post hoc comparisons test. All data are presented as mean ± SEM.

To examine how spatial exploratory behavior is altered in AD, we first characterized the exploratory patterns of 11‐month‐old AD and WT mice. We focused on two specific spatial exploratory behaviors: rearing and sniffing (Figure 1A). Compared to WT mice, AD mice displayed a fragmented exploratory pattern (Figure 1B), characterized by a higher frequency of spatial exploration (Figure 1C) but significantly shorter exploration durations per trial (Figure 1D). Principal component analysis (PCA) of these metrics revealed a distinct separation between the behavioral patterns of the two groups (Figure 1B). However, the total time spent in spatial exploration did not differ significantly between the groups (Figure 1E). Importantly, this fragmented pattern was not due to general deficits in locomotion or anxiety. An OFT showed no significant differences between AD and WT mice in total distance traveled (Figure 1F,G), straight‐line movement speed (Figure 1H), or time spent in the center zone (Figure 1I).

**FIGURE 2:**
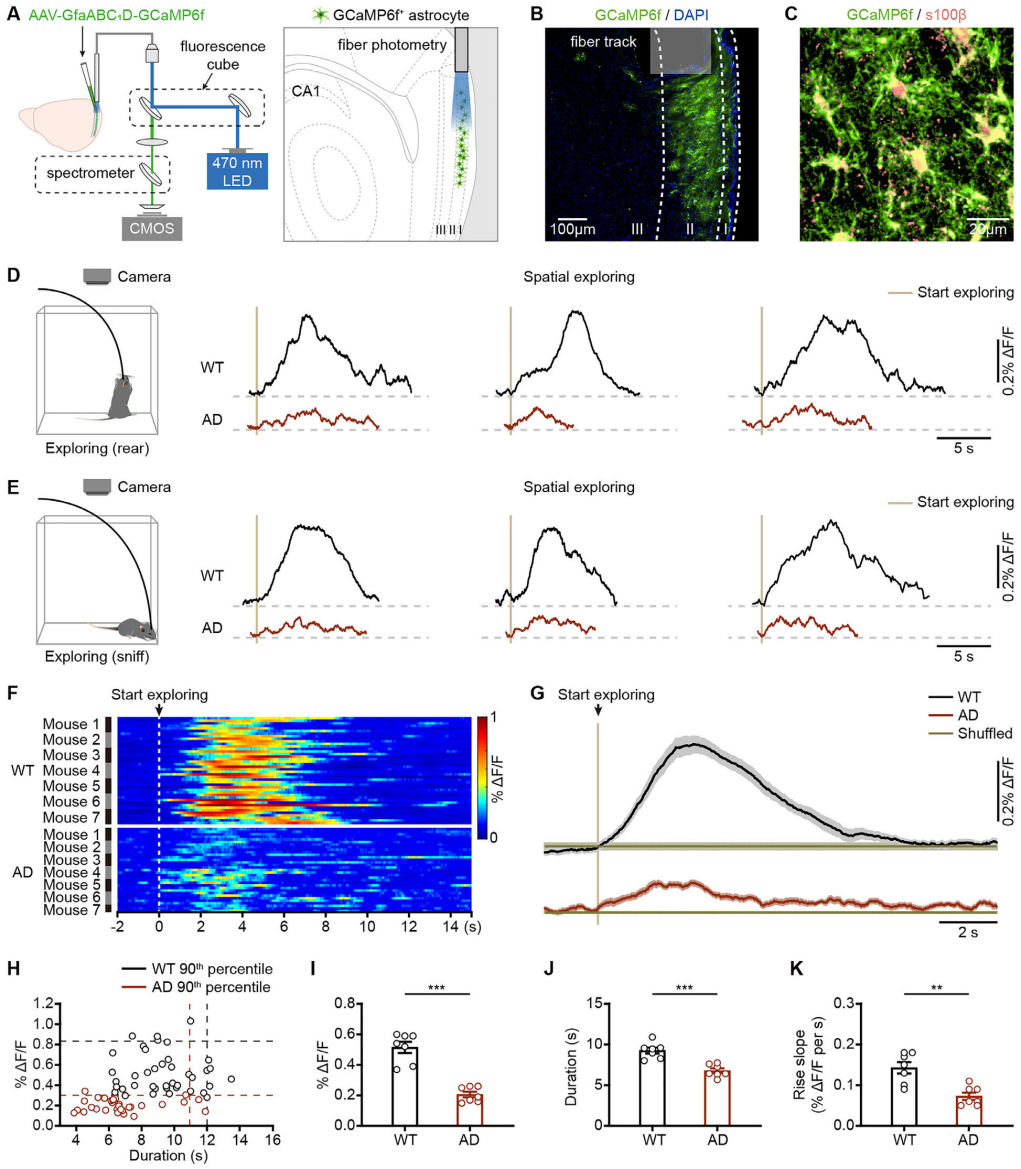
Astrocytic Ca^2+^ transients associated with reduced spatial exploration in the MECII of AD mice. (A) Left: AAV‐GfaABC_1_D‐GCaMP6f was stereotactically injected into the MECII to label astrocytes with GCaMP6f. Middle: Schematic illustrating the fiber photometry setup utilized to record astrocytic Ca^2+^ transients. Right: Fiber photometry recording site is positioned above the GCaMP6f‐labeled astrocytes within the MECII. (B) Representative histological image illustrating GCaMP6f‐labeled astrocytes situated beneath the fiber track in the MECII. (C) Representative high‐magnification confocal image showing co‐localization of GCaMP6f (green) with S100β (flesh). (D–E) Simultaneous recordings of astrocytic Ca^2+^ transients alongside spatial exploration behaviors during rearing (D, left) and sniffing (E, left). Representative trials of spatial exploration‐evoked astrocytic Ca^2+^ transients for WT and AD mice during rearing (D, right) and sniffing (E, right). Beige bars indicate exploration onsets. (F) Color map displaying spatial exploration‐evoked astrocytic Ca^2+^ transient amplitudes in the MECII of WT and AD mice (n = 42 trials from 7 mice in the WT group, n = 33 trials from 7 mice in the AD group). (G) Average astrocytic Ca^2+^ transients evoked by spatial exploration in the MECII of WT and AD mice (*n* = 42 trials from 7 mice in the WT group, n = 33 trials from 7 mice in the AD group). Beige bars represent exploration onsets. Sage green trace represents shuffled control. Shaded areas indicate SEM. (H) Scatterplot showing relationship between duration and peak amplitude of astrocytic Ca^2+^ transients. (I–K) Histograms summarizing amplitude (I), duration (J), and rise slopes (K) of astrocytic Ca^2+^ transients in two groups of mice (*n* = 7 mice per group). **p < 0.01, ***p < 0.001, independent samples via the Wilcoxon rank sum test. All data are presented as mean ± SEM.

Our previous study demonstrated that spatial exploration activates astrocytes in the MECII [12], a brain region essential for spatial coding [38, 39, 40, 41, 42]. These calcium (Ca^2^^+^) transients in MECII astrocytes, which are linked to spatial exploration, play a critical role in activity‐dependent plasticity in MECII neurons and in sustaining spatial exploratory behavior [12]. To investigate the cellular mechanisms underlying the fragmented spatial exploratory behavior observed in AD mice, we next examined astrocytic activity in the MECII. We used in vivo fiber photometry to record Ca^2^^+^ transients in MECII astrocytes specifically expressing GCaMP6f, a genetically encoded calcium indicator (Figure 2A). Post‐hoc histological analysis confirmed both accurate optic fiber placement (Figure 2B) and specific GCaMP6f expression in astrocytes, as indicated by S100β labeling (Figure 2C). The AAV‐GfaABC1D‐GCaMP6f‐based labeling strategy employed in our previous studies has been further validated in the present analysis [37, 43]. Our quantitative co‐labeling analysis revealed that 94.09 ± 1.21% of GCaMP6f‐expressing cells were positive for S100β, while 90.40 ± 1.17% of S100β‐positive astrocytes expressed GCaMP6f in the MECII (n = 6 mice). These findings confirm the high specificity of astrocyte labeling and the broad labeling efficiency of GCaMP6f.

Interestingly, while freely moving WT mice exhibited prominent astrocytic Ca^2^^+^ transients tightly synchronized with the onset of spatial exploration, AD mice showed a significant reduction in these transients during spatial exploration (Figure 2D,E). The decrease in astrocytic Ca^2^^+^ transients in AD mice was consistent across multiple trials, as evidenced by the heatmap (Figure 2F) and averaged peri‐event traces (Figure 2G). Quantitative analysis further confirmed that these reduced astrocytic Ca^2^^+^ transients in AD mice had distinct amplitude‐duration profiles, characterized by smaller amplitudes, shorter durations, and slower rise slopes compared to WT mice (Figure 2H–K). Moreover, in WT mice, the Ca^2^^+^ transients associated with spatial exploration were significantly larger than those recorded during other behaviors such as freely moving, eating, drinking, running, or grooming. In contrast, no significant differences were observed between spatial exploration‐related and other behaviors in AD mice (Figure 1J). These findings suggest that the fragmented spatial exploratory behavior in AD mice is likely related to reduced astrocytic Ca^2^^+^ transients in the MECII, which may impair the ability to sustain continuous spatial exploration.

### 2.2 Engrafted GPCs Migrate and Differentiate Into Astrocytes and Maintain a Youthful Morphology Within MECII of AD Brains

**FIGURE 3:**
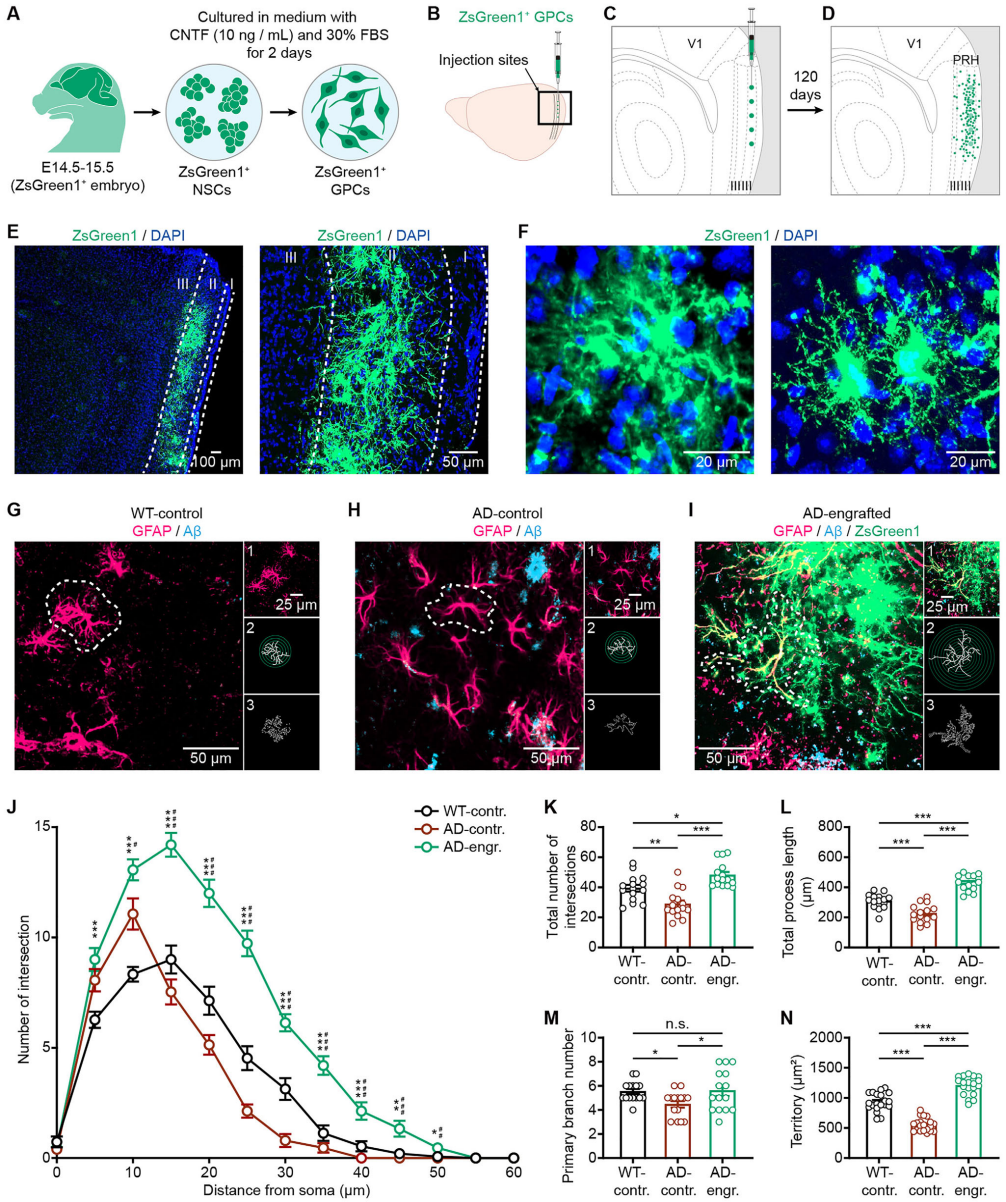
Engrafted GPCs can differentiate into astrocytes and maintain youthful morphology in the MECII of AD mice. (A) Schematic illustrating the generation procedure for GPCs. NSCs expressing ZsGreen1 are isolated from E14.5‐15.5 day embryos, expanded as neurospheres, and subsequently differentiated into GPCs. (B–D) Schematics illustrating transplantation and distribution of engrafted cells. A multi‐site injection strategy was employed for the transplantation of GPCs into the MEC (B). Coronal view indicating injection sites for GPC transplantation within the MECII of AD mice (C). Representative dot map showing the distribution of ZsGreen1^+^ engrafted astrocytes within MECII at 4 months post‐transplantation (D). (E–F) Representative confocal images illustrating engrafted astrocytes. At 4 months post‐transplantation, ZsGreen1^+^ GPCs (green) differentiate into astrocytes and integrate into the MEC (E). High‐magnification image revealing intricate fine structures of the engrafted astrocytes (F). (G–I) Confocal images of representative astrocytes from WT‐control (G), AD‐control (H), and AD‐engrafted (I) mice. Endogenous astrocytes are labeled with GFAP in magenta (G–I), while engrafted cells express ZsGreen1 in green (I). Right insets display the selected astrocyte outlined within the dashed line in the left image (**G**:1, **H**:1, **I**:1). Corresponding skeletonized rendition for Sholl analysis (5 µm inter‐shell distance, **G**:2, **H**:2, **I**:2) and respective astrocytic territory (**G**:3, **H**:3, **I**:3) are also included. (J) Sholl analysis for individual astrocytes illustrating the number of intersections between astrocytic branches and branchlets with concentric spheres centered on the cell soma (*n* = 15 cells from 5 mice per group). (K–N) Histograms summarizing total number of intersections (K), total process length (L), number of primary branches (M), and individual astrocyte territories (N) (K–M, n = 15 cells from 5 mice per group; N, n = 30 cells from 5 mice per group). *p < 0.05, **p < 0.01, and ***p < 0.001, via one‐way ANOVA with Bonferroni post hoc comparisons test. All data are presented as mean ± SEM.

Previous studies have suggested that astrocyte transplantation could provide a promising therapeutic approach for various neurological disorders [21, 23, 25, 27, 28, 29]. Given the observed deficits in astrocytic function during spatial exploration in AD mice, we sought to determine whether engrafted astrocytes in the MECII could replace dysfunctional astrocytes and alleviate the spatial exploratory deficits associated with AD. GPCs, a lineage‐restricted population of glial progenitors, may offer a more effective solution for treating glial‐related disorders [44]. Building on our previous work [33, 45], we developed a robust protocol to generate high‐purity GPCs from ZsGreen1^+^ embryonic NSCs (Figure 3A; Figure S1A). These NSCs uniformly expressed the NSC marker Nestin when cultured as neurospheres (Figure S1B). After induction, 95.00% of the cells expressed the GPC marker A2B5 (Figure S1C,D), confirming their identity before transplantation. Additional experiments showed that these GPCs could differentiate into astrocytes (Figure S1E,F).

In prior research, we demonstrated that engrafted GPCs successfully integrated into the neocortex of both adult [45] and aged animals [33]. However, it remained unclear whether these engrafted GPCs could migrate, differentiate, and integrate long‐term within the MECII of AD mice. In the current study, we transplanted GPCs into the MECII of 7–8‐month‐old AD mice, which were sacrificed 4 months post‐transplantation for histological analysis (Figure 3B–D). Our results revealed that ZsGreen1^+^ engrafted cells exhibited extensive survival and migration (Figure 3D). At 4 months post‐transplantation, approximately 7.1 × 10^3^ ZsGreen1^+^ GPC‐derived cells were found to survive per mouse, yielding an engraftment efficiency of approximately 0.59% (n = 6). This rate is slightly lower than the approximately 1% reported in healthy adult mice in our previous study [45]. This discrepancy is likely attributable to differences in host microenvironments, including factors related to age and pathological state. A significant portion of these cells migrated over 50 µm from the injection sites (Figure S2). Post‐hoc histological examination confirmed that the engrafted cells were primarily located in the MECII (Figure 3E), where they had differentiated into astrocytes with a complex, stellate morphology (Figure 3F).

Astrocytes in AD brains display both atrophic and reactive phenotypes, which vary depending on the disease stage and brain region [15]. The MEC is particularly vulnerable in AD [13, 14]. Studies have shown that astrocytes in the aging MEC exhibit fewer primary processes and a significant reduction in secondary branches [46]. To determine if engrafted astrocytes in MECII undergo similar morphological changes, we performed Sholl analysis on astrocytes from WT‐control, AD‐control, and AD‐engrafted groups. Our results revealed that resident astrocytes in AD mice (AD‐control) display a marked atrophic phenotype compared to WT mice (WT‐control, Figure 3G,H). These atrophic astrocytes exhibited a significant decrease in the number of intersections (Figure 3J,K), total process length (Figure 3L), primary branch number (Figure 3M), and territorial area (Figure 3N). In contrast, engrafted ZsGreen1^+^ astrocytes in the AD‐engrafted group showed a complex, highly branched morphology four months post‐transplantation (Figure 3I). Quantitative analysis confirmed that engrafted astrocytes in AD mice retained a youthful morphology, with increased intersections (Figure 3J,K), longer process length (Figure 3L), more primary branches (Figure 3M), and larger territorial area (Figure 3N) compared to resident astrocytes in age‐matched WT mice. These findings suggest that engrafted astrocytes can preserve a healthy morphology and integrate long‐term into the MECII of AD brains.

### 2.3 Engrafted Astrocytes Re‐Establish Perivascular AQP4 Polarization and Reduce Aβ Burden in the MECII of AD Brains

AQP4, a water channel primarily located on the perivascular endfeet of astrocytes [47, 48], plays a critical role in the clearance of Aβ and abnormal tau [49]. Reduced polarized expression of AQP4 has been implicated in AD pathology [50], and its polarized distribution is closely linked to the astrocyte phenotype [49]. Having established that engrafted astrocytes maintain a youthful morphology in the MEC of AD brains, we subsequently investigated whether these engrafted astrocytes could enhance AQP4 polarization and alleviate Aβ burden.

**FIGURE 4:**
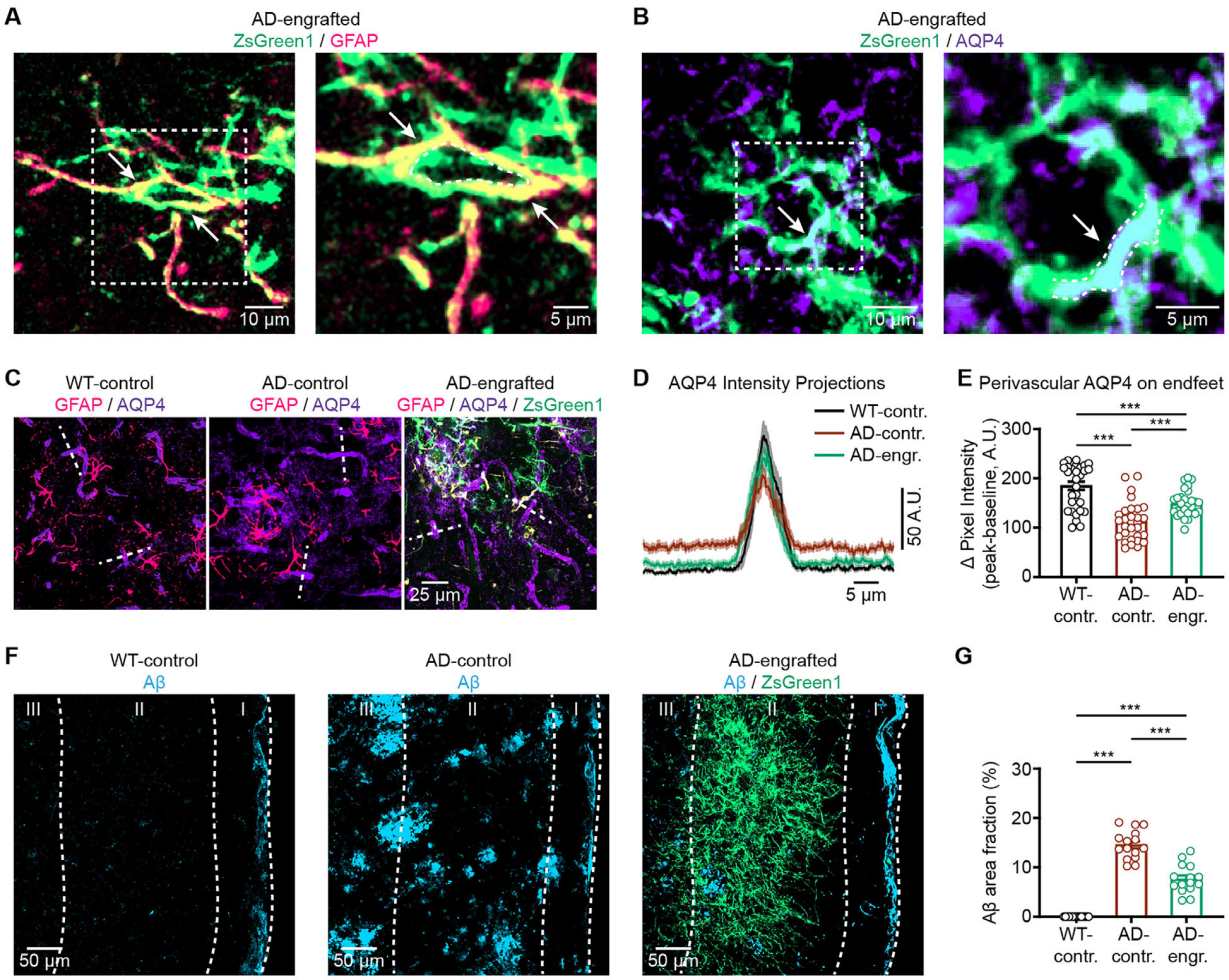
Engrafted astrocytes re‐establish perivascular AQP4 polarization and reduce Aβ burden in the MECII of AD mice. (A,B) Engrafted astrocytes form AQP4‐expressing perivascular endfeet. (A) Representative confocal image showing ZsGreen1^+^ engrafted astrocytic processes (green) and GFAP^+^ astrocytic structures (magenta) forming perivascular endfoot‐like structures at 4 months post‐transplantation in the MECII of AD mice. Right panel presents a higher magnification of the boxed region. (B) Immunostaining demonstrates co‐localization of AQP4 (purple) with perivascular endfeet of engrafted astrocytes (ZsGreen1^+^, green). Right panel presents a higher magnification of the boxed region. (C) AD‐control of MECII (middle) exhibits a loss of perivascular AQP4 polarization compared to the well‐polarized state of AQP4 in the WT‐control of MECII (left). However, MECII from AD mice transplanted with engrafted astrocytes (ZsGreen1^+^, right) retains AQP4 polarization. (D) AQP4 immunofluorescence assessed in a linear region of interest extending outward from the vessel (dashed lines in panel C, n = 30 vessels per group from 6 mice). (E) Histograms summarizing perivascular AQP4 expression (peak‐baseline) indicating that AQP4 expression was significantly elevated in perivascular MECII of AD‐engrafted mice compared to AD‐control mice (n = 30 vessels per group from 6 mice). (F,G) Astrocyte engraftment significantly reduces Aβ plaque burden in the MECII. Representative images depicting Aβ (cyan) immunofluorescence across three groups of mice (F). Histograms summarizing Aβ area fraction in the MECII for the three groups of mice (*n* = 15 brain slices per group from 5 mice, G). ***p < 0.001, via one‐way ANOVA with Bonferroni post hoc comparisons test. All data are presented as mean ± SEM.

High‐resolution confocal imaging revealed that the processes of engrafted ZsGreen1^+^ astrocytes formed extensive perivascular endfeet that tightly ensheathed blood vessels in the MECII of AD mice (Figure 4A). These newly formed endfeet expressed AQP4, as evidenced by its co‐localization with ZsGreen1^+^ processes (Figure 4B). Additional CD31/AQP4/ZsGreen1 staining further reveals that ZsGreen1^+^ engrafted astrocytic processes envelop host CD31^+^ blood vessels, forming AQP4‐positive perivascular endfoot‐like structures within the MECII (Figure S3). These findings support a direct structural interaction between engrafted astrocytes and the host glial‐vascular unit. To assess whether engrafted astrocytes could restore impaired AQP4 polarization, we compared AQP4 distribution across groups. In WT‐control mice, AQP4 was sharply polarized to the perivascular endfeet (Figure 4C, left). In contrast, AQP4 expression was diffuse and poorly polarized in the AD‐control group (Figure 4C, middle). In the AD‐engrafted group, however, AQP4 polarization was significantly improved (Figure 4C, right). Linescan analysis of blood vessels confirmed a sharp peak in AQP4 intensity at the perivascular space in both WT‐control and AD‐engrafted mice, which was absent in AD‐control mice (Figure 4D). Quantification of peak intensity further demonstrated that astrocyte engraftment restored the AQP4 polarization in AD‐engrafted mice (Figure 4E). To further clarify the distinct roles of host and engrafted astrocytes in AQP4 repolarization, we classified the perivascular AQP4 polarization regions into two groups: AD‐host regions without engrafted astrocyte processes, and AD‐engrafted regions containing these processes (Figure S4). AD‐engrafted regions exhibited significantly higher AQP4 polarization compared to AD‐control regions, while AD‐host regions showed no significant changes (Figure S4). This indicates that engrafted astrocytes, rather than host astrocytes, primarily mediate the AQP4 repolarization.

Given that proper AQP4 polarization is crucial for the clearance of interstitial solutes, such as Aβ, we next assessed the Aβ plaque burden in the MECII. Consistent with the restoration of AQP4 polarization, we observed a significant reduction in Aβ plaque load in AD‐engrafted mice compared to AD‐control mice (Figure 4F). Quantitative analysis of the Aβ‐positive area fraction confirmed that astrocyte engraftment substantially reduced the plaque burden in the MECII (Figure 4G).

Furthermore, to investigate the glymphatic flux‐based evidence linking perivascular AQP4 polarization to Aβ clearance, we performed an intracisterna magna injection of a fluorescent cerebrospinal fluid (CSF) tracer to label glymphatic flux (Figure S5A). The CSF tracer signal was detected along CD31^+^ blood vessels and adjacent to AQP4^+^ perivascular domains in the MECII (Figure S5B). We further quantified the Aβ‐positive area fraction across distance bins from tracer‐positive regions and found that Aβ deposition increased with distance from the center of the CSF tracer‐positive area (Figure S5C,D). These results indicate that Aβ can be cleared by glymphatic flux through perivascular AQP4. Consequently, the reduced Aβ plaque burden in the MECII of AD‐engrafted mice (Figure 4F,G) may be attributed to the enhanced AQP4 polarization induced by engrafted astrocytes (Figure 4A–E).

### 2.4 Engrafted Astrocytes Alleviate Local Neuroinflammation and Reduce Microglial Activation in the MECII of AD Brains

**FIGURE 5:**
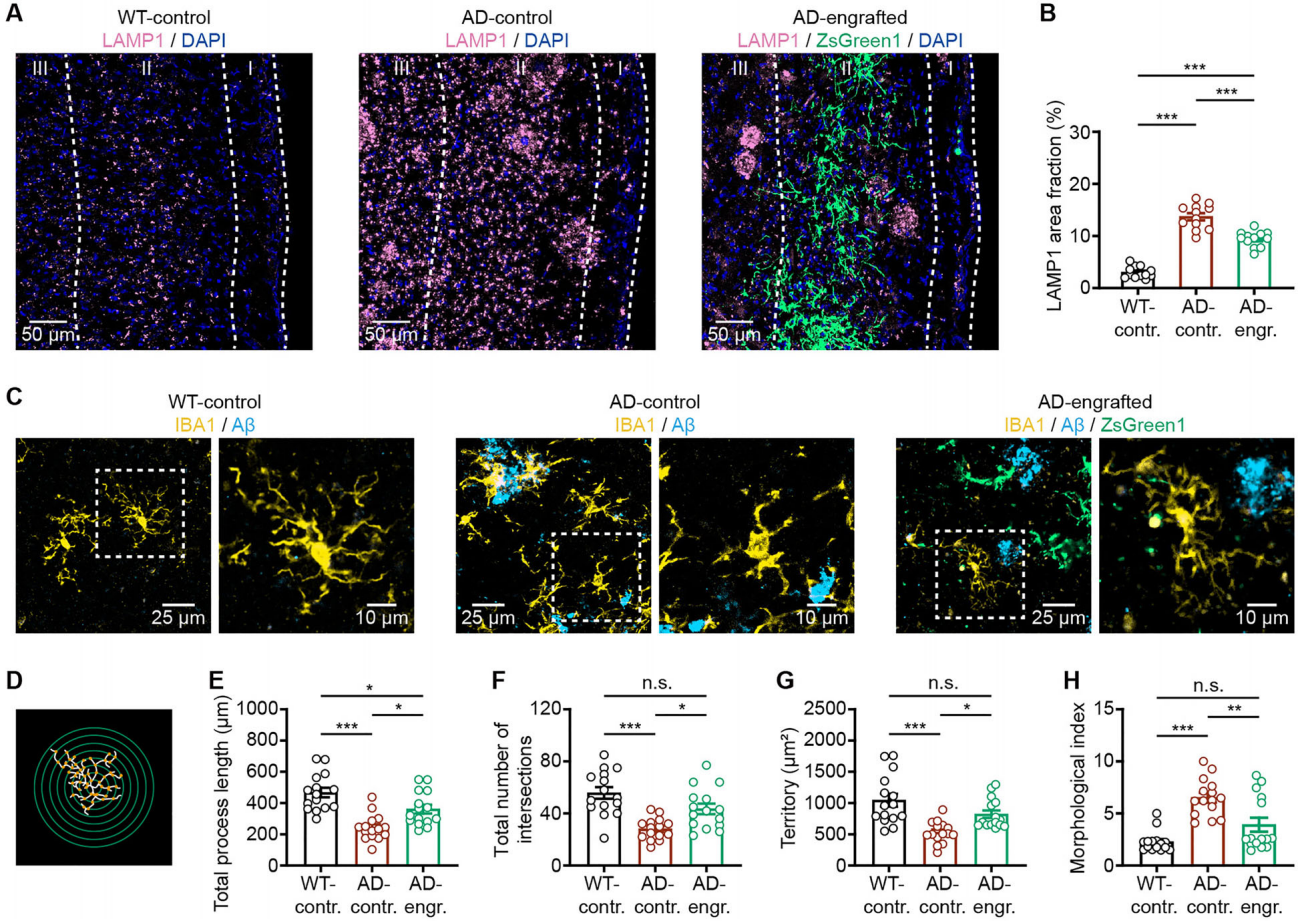
Engrafted astrocytes alleviate inflammation and promote a homeostatic microglial phenotype in AD mice. (A,B) Engrafted astrocytes alleviate LAMP1‐associated pathology in MECII. Representative immunofluorescence images illustrating LAMP1 (pink, marker linked to dystrophic neurites and inflammation) in WT‐control, AD‐control, and AD‐engrafted mice (A). Histograms summarizing LAMP1 area fraction in MECII across three groups of mice (*n* = 12 brain slices per group from 5 mice, B). (C) Immunofluorescence staining co‐labeling IBA1 (yellow), Aβ (cyan), and engrafted astrocytes (ZsGreen1^+^, green) in MECII of three groups of mice. Images on the right of each group represent enlarged versions of the left images. (D–H) Quantification of microglial morphology confirming a transition toward a homeostatic state. A schematic representation of the Sholl analysis is provided to assess the complexity of microglial processes. Orange dots indicate intersection points at a 5 µm inter‐shell distance (D). Histograms summarizing key morphological parameters: total process length (E), total number of intersections (F), cellular territory (G), and morphological index (H) (*n* = 15 cells per group from 5 mice). *p < 0.05, **p < 0.01, ***p < 0.001, via one‐way ANOVA with Bonferroni post hoc comparisons test. All data are presented as mean ± SEM.

Given that a reduced Aβ plaque burden is often associated with an improved neuroinflammatory environment [51, 52, 53, 54, 55, 56], we subsequently examined whether the engrafted astrocytes were accompanied by a reduction in neuroinflammation within the MEC of AD mice. To assess Aβ‐induced neuroinflammation, we first confirmed the spatial correlation between Aβ plaques and LAMP1, a marker of neuroinflammation [57, 58, 59, 60, 61] and lysosomal activity [62, 63, 64, 65, 66, 67, 68], in the MEC of AD brains (Figure S6A). Our results demonstrated a clear spatial association between LAMP1 and Aβ plaques in both the AD‐control and AD‐engrafted groups (Figure S6). Subsequently, we quantified the LAMP1 area fraction across all three groups. LAMP1 expression was significantly higher in AD‐control mice compared to WT‐controls (Figure 5A,B). In contrast, astrocyte engraftment resulted in a marked reduction of the LAMP1‐positive area fraction in the AD‐engrafted group compared to the AD‐control group (Figure 5A,B). These findings suggest that engrafted astrocytes are associated with reduced Aβ‐related neuroinflammation in the MEC of AD mice.

Previous studies have shown that Aβ‐induced neuroinflammation triggers microglial activation [69, 70, 71, 72]. We next investigated whether engrafted astrocytes, by improving the neuroinflammatory environment, could reduce microglial activation in the MEC of AD mice. In WT control mice, microglia exhibited a homeostatic, ramified morphology. In contrast, microglia surrounding Aβ plaques in AD control mice displayed a classic activated, amoeboid morphology with retracted processes (Figure 5C, left and middle). Remarkably, in AD‐engrafted mice, microglia reverted to a homeostatic, ramified morphology characterized by extensive, fine processes (Figure 5C, right). Sholl analysis revealed that microglia in the AD control group had reduced total process length, fewer intersections, and smaller territorial coverage, while exhibiting an increased morphological index (Figure 5D–H). In contrast, astrocyte engraftment in the AD‐engrafted group reversed these morphological changes, restoring microglial structure to a more homeostatic state (Figure 5D–H). These findings suggest that engrafted astrocytes mitigate microglial activation in the MEC of AD brains by improving the local neuroinflammatory environment.

### 2.5 Engrafted Astrocytes Reduce Overexpression of CX43 and D‐Serine, and Reverse Synapse Loss in the MECII of AD Brains

**FIGURE 6:**
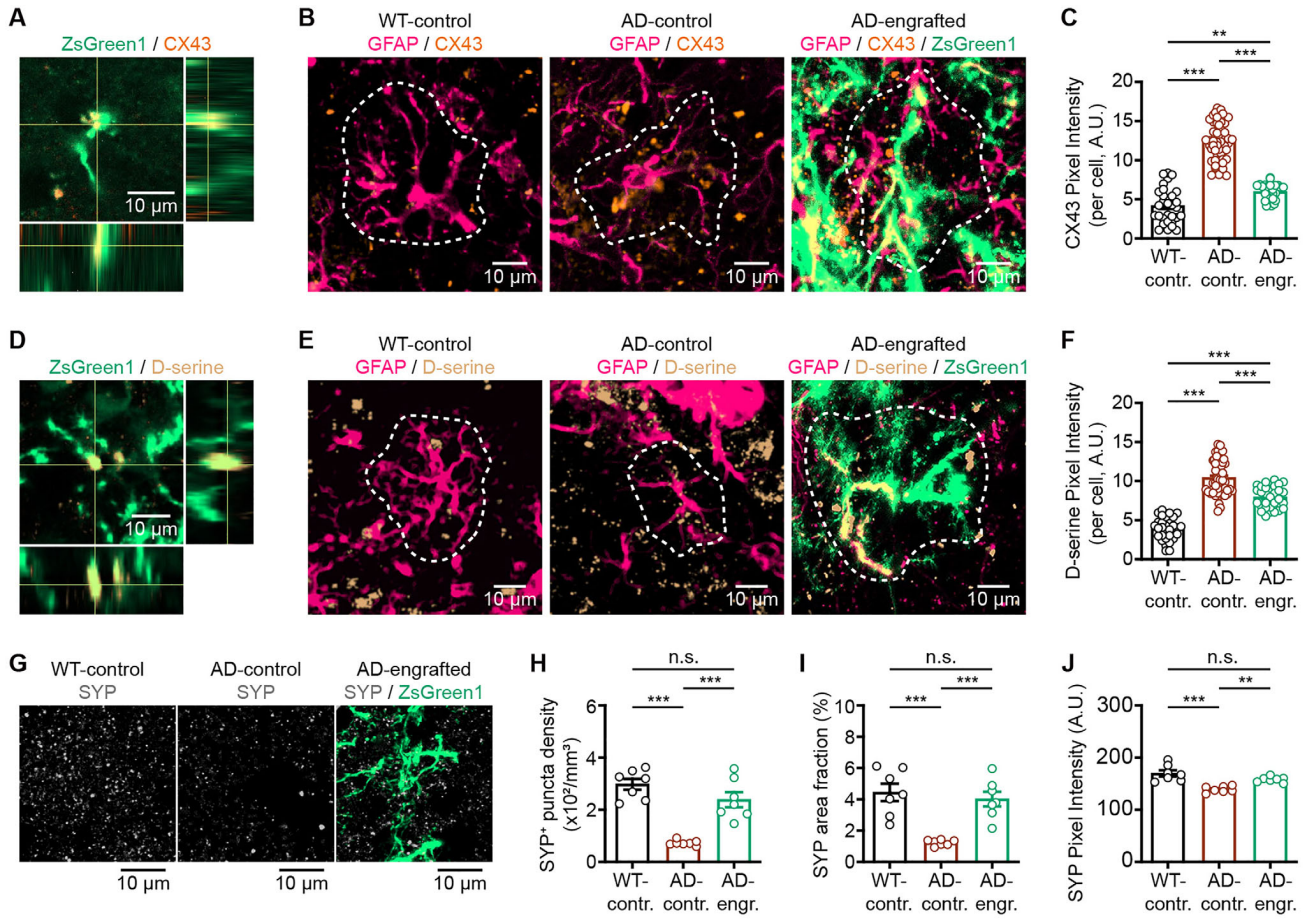
Engrafted astrocytes reduce CX43 and D‐serine overexpression and preserve synaptic markers in AD mice. (A) Orthogonal views from Z‐stack confocal images confirming that engrafted astrocytes (ZsGreen1^+^, green) express CX43 (orange). (B) Representative images illustrating CX43 immunofluorescence within individual astrocytic domains (outlined) in the MECII of WT‐control, AD‐control, and AD‐engrafted mice. (C) Histograms summarizing quantification of CX43 fluorescence intensity per astrocytic domain for three groups of mice (*n* = 30 cells from 6 mice per group). (D) Orthogonal views from Z‐stack confocal images confirming that engrafted astrocytes (ZsGreen1^+^, green) express D‐serine (gold). (E) Representative images of D‐serine immunofluorescence within outlined astrocytic domains in three groups of mice. (F) Histograms summarizing quantification of D‐serine fluorescence intensity per astrocytic domain for three groups of mice (n = 30 cells from 6 mice per group). (G) Representative images of the presynaptic marker SYP (gray) in the MECII region of three groups. (H–J) Histograms summarizing density of SYP‐positive puncta (H), SYP‐positive area fraction (I), and average SYP fluorescence intensity (J) (*n* = 7 mice per group). **p < 0.01, ***p < 0.001, via one‐way ANOVA with Bonferroni post hoc comparisons test. All data are presented as mean ± SEM.

Astrocytes communicate with each other through gap junctions composed of connexins (Cxs) [73], forming interconnected networks. Previous research has shown that connexin 43 (CX43) expression is specifically upregulated around Aβ plaques [74, 75], where it mediates enhanced Ca^2+^ waves and ATP release in astrocytes [76, 77], contributing to the progression of AD pathology [77]. To assess whether engrafted astrocytes were associated with reduced CX43 expression in the MEC, we examined the levels of CX43 within astrocytic territories in this region. As expected, our results confirmed that astrocytic CX43 expression was significantly elevated in the AD‐control group compared to the WT‐control group (Figure 6A–C), in line with previous reports [74, 75]. However, in the AD‐engrafted group, CX43 expression was markedly reduced compared to the AD‐control group (Figure 6A–C). These findings suggest that engrafted astrocytes are associated with reduced CX43 expression in AD brains, potentially contributing to the alleviation of pathological network alterations.

Astrocytic D‐serine production has been implicated in the pathogenesis of AD [78]. Elevated Aβ levels trigger the release of D‐serine from reactive astrocytes, which in turn contributes to neuronal excitotoxicity and cell death in AD brains [79]. Reducing the neurotoxic release of D‐serine from astrocytes could therefore provide a potential therapeutic strategy for AD [80]. To examine this, we investigated whether engrafted astrocytes were associated with reduced D‐serine expression and preserved neuronal synapses in the MEC. Consistent with prior studies [80], our results showed increased D‐serine expression in astrocytic territories (Figure 6D–F) and a corresponding decrease in synapse density, as indicated by SYP staining, in the AD‐control group compared to WT‐controls (Figure 6G–J). In contrast, in the AD‐engrafted group, D‐serine expression within astrocytic territories was significantly reduced (Figure 6D–F), and synapse density was restored (Figure 6G–J) compared to the AD‐control group. These results suggest that engrafted astrocytes are associated with a reduction in D‐serine overexpression and the preservation of synaptic integrity in the MEC of AD brains.

### 2.6 Engrafted Astrocytes Within the MECII Rescue Fragmented Spatial Exploration of AD Mice

**FIGURE 7:**
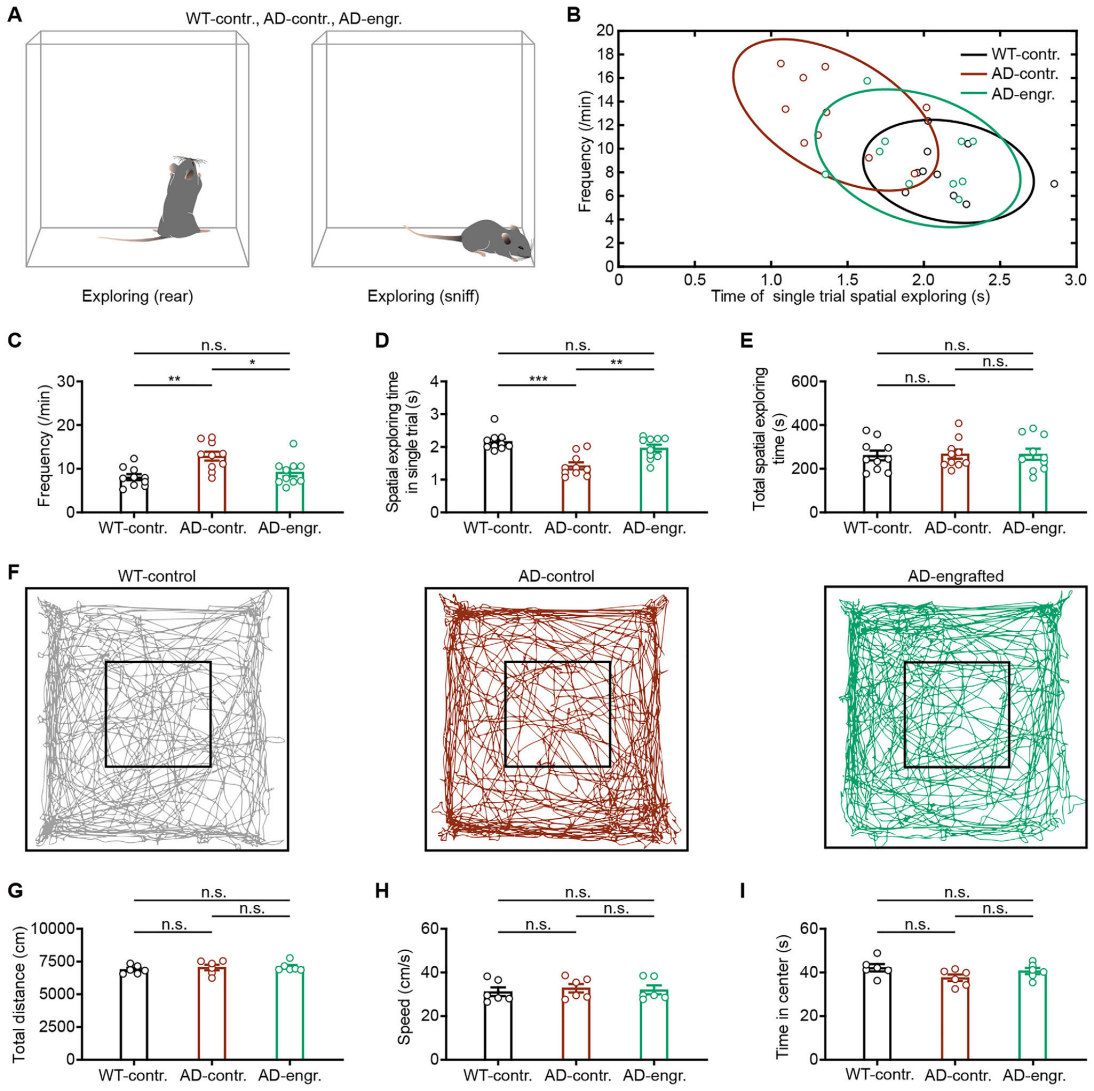
Engrafted astrocytes within the MECII restore impaired spatial exploration in AD mice. (A) Schematics illustrating spatial exploration behaviors observed in WT‐control, AD‐control, and AD‐engrafted mice during rearing and sniffing. (B) Principal component analysis on the exploration frequency and single exploration time in WT‐control, AD‐control, and AD‐engrafted mice (n = 10 mice per group). (C–E) Histograms summarizing frequency (C), duration of single trials (D) and total time spent in spatial exploration (E) for three groups of mice (n = 10 mice per group). (F) Representative trajectory plots of OFT in three groups of mice. Black box represents the central region of the arena. (G–I) Histograms summarizing total distance (G), locomotion speed (H), and time spent in the central area (I) during OFT across three groups of mice (n = 6 mice per group). *p < 0.05, **p < 0.01, ***p < 0.001, via one‐way ANOVA with Bonferroni post hoc comparisons test. All data are presented as mean ± SEM.

Our previous study demonstrated that astrocytes in the MECII are crucial for spatial exploration [12]. The present study builds on these findings, showing that impaired spatial exploration in AD mice correlates with reduced astrocytic Ca^2+^ transients in MECII (Figures 1 and 2). Engrafted astrocytes exhibited a youthful morphology (Figure 3) and were associated with a reduced Aβ burden (Figure 4), attenuated neuroinflammation (Figure 5), and restored synapse density (Figure 6) in the MECII of AD brains. Based on these findings, we next aimed to assess whether astrocyte engraftment in MECII could rescue the fragmented spatial exploratory behaviors observed in AD mice. In the behavioral analysis, we evaluated spatial exploration by measuring rearing and sniffing frequencies in WT‐control, AD‐control, and AD‐engrafted groups (Figure 7A). Consistent with earlier behavioral results (Figure 1), AD‐control mice exhibited a fragmented exploration pattern, characterized by increased frequency and shorter duration of exploration compared to WT‐controls (Figure 7B–D).

Remarkably, four months after transplantation, AD‐engrafted mice displayed a significant decrease in exploratory frequency and an increase in exploratory duration (Figure 7B–D). However, the total time spent on spatial exploration remained similar across all three groups (Figure 7E). To ensure that improvements in spatial exploration were not influenced by changes in general activity or anxiety, we subjected all groups to the OFT. The analysis revealed no significant differences in total distance traveled, straight‐line movement speed, or time spent in the center zone between the WT‐control, AD‐control, and AD‐engrafted groups (Figure 7F–I).

To further address a potential olfactory confound in sniffing‐related exploration, we conducted a buried food test. Both AD‐control and AD‐engrafted mice exhibited longer latencies in locating the buried food pellet compared to WT‐control mice; however, no significant difference was observed between the AD‐control and AD‐engrafted groups (Figure S7). These findings suggest that astrocyte engraftment in MECII did not restore olfactory function.

To determine whether the observed behavioral improvements are attributable to the astrocyte‐specific properties of the engrafted cells, we examined AD mice at 0.5 months post‐GPC transplantation. At this time point, only 0.57% ± 0.08% of ZsGreen1^+^ grafts expressed GFAP, indicating that most GPCs remained undifferentiated (Figure S8A,B). AD‐engr‐0.5 M mice exhibited spatial exploration comparable to AD controls, while AD‐engr‐4 M mice demonstrated decreased exploration frequency and longer single‐trial duration (Figure S8C,D). Notably, total exploration time did not reveal any significant intergroup differences (Figure S8E). These findings indicate that the behavioral rescue observed in AD‐engr‐4 M mice arises from astrocyte‐specific properties rather than non‐specific graft effects.

Building on previous evidence that MEC astrocytes are essential for spatial exploration [12], we further investigate whether engrafted astrocytes within the MECII integrate into host neural circuits during exploratory behavior. In this study, we analyze c‐Fos expression in engrafted astrocytes located in the MECII of AD mice following spatial exploration (Figure S9). Compared to mice exposed to their home cage, the proportion of engrafted astrocytes labeled with c‐Fos significantly increased following spatial exploration (Figure S9). This finding provides evidence related to functional activity, indicating that these engrafted astrocytes have integrated into the host neural circuits and were recruited during exploratory behavior in AD mice.

To investigate whether other cognitive functions can be rescued following MECII astrocyte transplantation, we conducted two spatial memory behavioral assays: the Y‐maze novel‐arm test and the novel location recognition test. Compared to the AD‐control group, AD mice that received MECII astrocyte transplantation (AD‐engrafted group) spent mildly more time in the novel arm (Figure S10A–C) and exhibited a significantly elevated object location recognition index (Figure S10D–F). These findings suggest that MECII astrocyte transplantation effectively restores impaired spatial memory in AD mice.

These results suggest that astrocyte engraftment in MECII restores fragmented spatial exploration in AD mice, without affecting locomotor activity, anxiety‐like behaviors, or olfactory‐guided food seeking.

## 3 Discussion

Impaired spatial exploratory behavior is a hallmark feature of AD [4, 5, 6, 7]. Previous studies have shown that astrocytes in the MEC play a key role in facilitating spatial exploration [12], but these cells undergo both morphological and functional alterations in AD [81]. The engraftment of astrocytes has yielded promising outcomes in various neurological disorders, including PD [23, 24], HD [27], TBI [29, 30], and ischemic events [28]. Building on this, the present study investigates whether engrafted astrocytes in the MEC can improve the pathological environment in AD brains and rescue disrupted spatial exploratory behaviors. Our findings demonstrate that spatial exploration in AD mice is fragmented and correlates with reduced astrocytic Ca^2^^+^ transients in the MECII. Engrafted astrocytes exhibit a youthful morphology, enhance perivascular AQP4 polarization, and are associated with a reduced Aβ burden, attenuated regional neuroinflammation, and recovered synapse density. These cellular and pathological improvements correlate with restored spatial exploration patterns in AD mice. This study makes a significant contribution to the field by highlighting the protective roles of engrafted astrocytes in the MEC of AD brains. It further demonstrates that these astrocytes enhance cognitive behaviors, particularly spatial exploration, offering a promising therapeutic approach for AD.

Animal behavior is influenced not only by neuronal activity but also by the coordinated function of astrocytes and neurons [82, 83]. The interaction between these two cell types plays a key role in regulating essential aspects of behavior, such as circadian rhythms, sleep‐wake cycles, feeding, sensory‐motor functions, and decision‐making [82]. Reduced astrocyte function at synapses has been linked to dysfunction in neuronal networks, contributing to cognitive impairments observed in AD [84]. In vivo studies have demonstrated that astrocytes in AD exhibit Ca^2+^ hyperactivity, including irregular oscillations and abnormal long‐range Ca^2+^ waves [15]. This phenomenon resembles the neuronal hyperexcitability commonly seen in AD mouse models [85, 86]. Additionally, astrocytes in another AD model showed an increased frequency of spontaneous oscillations even before amyloid plaques appeared [87]. In vitro studies further confirm that β‐amyloid exposure induces Ca^2+^ transients in cultured astrocytes [88, 89], and a higher frequency of these transients has been detected in slices from AD mice [90]. However, it remains unclear whether changes in astrocytic Ca^2+^ dynamics are specifically linked to behavioral disturbances in AD, or whether these alterations correlate with specific behavioral impairments in AD patients.

In line with previous studies [4, 5, 6, 7], our research confirms the impaired spatial exploratory behaviors observed in AD mice. These behaviors displayed a fragmented pattern, characterized by more frequent but shorter bouts of exploration during individual trials (Figure 1B–D). Notably, we show that astrocytic Ca^2+^ transients within the MECII, which are typically associated with spatial exploration, are significantly reduced in AD mice (Figure 2D–K). Our previous work has demonstrated that astrocytic Ca^2+^ transients in the MEC play a critical role in mediating local neuronal activity and supporting spatial exploration [12]. Based on this, we hypothesize that the reduction in astrocytic Ca^2+^ transients within MECII leads to dysfunctional astrocytic support for neuronal activities, contributing to the observed impairment of spatial exploratory behaviors in AD mice. These findings are crucial because they identify abnormal astrocytic activity as a key factor in cognitive behavior deficits associated with AD pathology. Future research should investigate the molecular and physiological processes that link Ca^2+^ transients in MECII astrocytes to deficits in exploratory behavior associated with Alzheimer's disease, considering both upstream and downstream interactions.

Astrocytes play an increasingly recognized role in the pathogenesis of neurodegenerative diseases, particularly AD [31]. In AD, astrocytic pathological remodeling disrupts their homeostatic and neuroprotective functions, representing a pivotal aspect of disease progression [17]. Evidence shows that both astrocytic reactivity and degeneration occur in AD, often preceding the hallmark histopathological features of the disease, such as amyloid plaques and neurofibrillary tangles [15, 91]. In this context, astrocyte degeneration and activation are closely linked to a decline in their homeostatic support functions, such as impaired glutamate uptake, which in turn contributes to synaptic damage and cognitive decline [79]. These pathological changes position astrocytes as promising targets for therapeutic intervention in AD [17]. Cell‐based therapies, an emerging treatment strategy, aim to promote tissue regeneration and restore cellular function [20]. Astrocytes are particularly well‐suited for such therapies due to their ability to interact with surrounding cells and regulate multiple aspects of cellular function within the central nervous system (CNS) [20]. Astrocyte engraftment is expected to influence not only the astrocytic network but also the interactions between other cell types [20].

Several studies have shown that engrafting astrocytes can slow disease progression and improve functional outcomes in translational models of ALS, PD, HD, ischemic injury, and traumatic CNS damage [20]. However, only a few studies have investigated the application of astrocyte engraftment to mitigate AD pathology. Research has demonstrated that engrafting focal neural precursor cells, which differentiate into glial cells, can rescue cortical neurons. This suggests that engrafted astrocytes may play a neuroprotective role in AD [34]. Engrafted astrocytes have been shown to internalize human Aβ immunoreactive material in vivo [25], but there is still a lack of comprehensive evidence regarding the extent to which transplanted astrocytes can alleviate AD pathology. Our findings demonstrate that engrafted astrocytes in AD mice exhibit significantly younger morphologies and more complex structures compared to the resident astrocytes in age‐matched WT mice (Figure 3). The engrafted astrocytes successfully integrated into the host AD brain and were associated with improved perivascular AQP4 polarization (Figure 4A–E), decreased Aβ plaque burden (Figure 4F,G), and attenuated regional neuroinflammation along with reduced microglial activation within the MECII of AD mice (Figure 5). Furthermore, the engrafted astrocytes were associated with a reduction in the overexpression of CX43 and D‐serine, as well as a recovery of synapse density in the MECII of AD brains (Figure 6).

The mechanism underlying the amelioration of AD pathology by engrafted astrocytes may be linked to their enhancement of the host microenvironment within the MEC, a region particularly vulnerable to AD pathogenesis. First, at the level of the glial‐vascular unit (GVU), perivascular AQP4 polarization is recognized as a critical driver of glymphatic and perivascular transport, as well as Aβ clearance [92, 93, 94, 95]. The depolarization of AQP4 in AD contributes to impaired glymphatic exchange and aggravated Aβ deposition [95, 96, 97]. Consistent with this framework, our study demonstrates that engrafted astrocytes restore perivascular AQP4 polarization (Figure 4A–E) and reduce Aβ plaque burden in the MECII of AD mice (Figure 4F,G). Supporting a causal link, supplementary data confirm that Aβ can be cleared through glymphatic flux via perivascular AQP4 (Figure S5). Collectively, these results suggest that the reduced Aβ burden in the MECII of engrafted AD mice is mediated, at least in part, by the engrafted astrocyte‐induced recovery of perivascular AQP4 polarization. Furthermore, the reduced Aβ deposition induced by engrafted astrocytes further alleviates local neuroinflammation (Figure 5A,B) and microglial activation (Figure 5C–H) in the MEC. In addition, the reduced expression of CX43 (Figure 6A–C) and D‐serine (Figure 6D–F), alongside the restoration of synaptic integrity (Figure 6G–J), suggests that the engrafted astrocytes also mitigate local microenvironmental neurotoxicity. This finding aligns with previous studies that have linked CX43 upregulation [98, 99] and excessive D‐serine release [80, 100] to synaptic loss and cognitive decline. Therefore, the cognitive benefits observed from astrocyte transplantation in AD mice may be partially attributed to the downregulation of CX43 and the reduction of D‐serine release (Figure 6A–F). These findings collectively support astrocyte transplantation as a viable proof‐of‐concept strategy for enhancing the local pathological microenvironment in a mouse model of AD.

Moreover, engrafted astrocytes have been found to mitigate behavioral impairments in a variety of brain disorders [21, 30, 101, 102]. In mouse models of HD, astrocyte engraftment into the striatum has been shown to slow both motor and cognitive degeneration, ultimately improving survival rates [101]. In the context of global ischemia, astrocyte engraftment enhances neuroprotection, resulting in improved behavioral outcomes [102]. In the context of ALS, astrocyte engraftment has been shown to slow the decline of forelimb motor skills and respiratory functions [21]. Similarly, astrocyte‐based therapies have led to significant motor function recovery in models of TBI [30]. However, whether engrafted astrocytes can reverse the behavioral deficits associated with AD remains unclear. This study aimed to evaluate the effects of astrocyte engraftment on behavioral outcomes in AD mice. Our results demonstrate that AD mice exhibit fragmented spatial exploratory behaviors (Figure 1). Importantly, engrafted astrocytes within the MECII region ameliorated these fragmented behaviors without negatively affecting locomotor ability or inducing anxiety‐like behaviors (Figure 7). This behavioral improvement is correlated with the long‐term survival of engrafted astrocytes in the MECII, enhanced AQP4 perivascular polarization, alleviated cerebral Aβ plaque burden, attenuated regional neuroinflammation, and the preservation of synapses. Future research will further dissect the distinct contributions of each underlying mechanism to the observed rescue of behavioral deficits.

Finally, APP/PS1 transgenic mice, a model predominantly associated with amyloid pathology in familial AD, were utilized in this study. This model is widely recognized in preclinical AD research for demonstrating robust amyloid plaque deposition, glial activation, neuroinflammation, and behavioral impairments [103, 104]. However, it does not fully replicate the complexities of human AD pathology, particularly with respect to tau lesions and the intricacies of sporadic AD [103, 105]. Future research will aim to validate the therapeutic efficacy of engrafted astrocytes across various complementary AD models, including the 3xTg‐AD model, which exhibits concurrent amyloid and tau pathologies [106], the rapidly progressive 5xFAD model [107], App knock‐in models that eliminate artifacts associated with APP overexpression [105], and astrocyte systems derived from induced pluripotent stem cells (iPSCs) of AD patients [108].

Collectively, our results provide proof‐of‐concept for behavioral validation and present a novel therapeutic framework for AD by demonstrating the translational potential of region‐targeted astrocyte transplantation.

## 4 Conclusions

In summary, our study demonstrates that astrocyte transplantation into the MEC, a region particularly vulnerable in AD, effectively restores a key deficit in spatial exploration in an AD mouse model. The engrafted astrocytes integrate into the host brain by re‐establishing perivascular AQP4 polarization, which facilitates enhanced Aβ clearance. This modification of the pathological microenvironment reduces local neuroinflammation and mitigates synaptic loss. These findings support a proof‐of‐concept role for region‐specific astrocyte replacement in improving deficits related to spatial exploration and in ameliorating the regional pathological microenvironment in AD mice.

## 5 Experimental Section

### 5.1 Animals

We used APP/PS1 transgenic mice, a model of AD, and wild‐type (WT) C57BL/6J mice for this study. Fluorescently labeled GPCs were obtained from ZsGreen1 reporter mice (CAG‐LoxP‐ZsGreen‐Stop‐LoxP‐td Tomato; T006163). All mouse lines were sourced from GemPharmatech (Nanjing, China). The mice were housed at 22–25°C with 50%–60% relative humidity, maintained on a 12‐h light‐dark cycle, and given ad libitum access to food and water. Mice implanted with optical fibers were kept individually. All procedures followed the guidelines set by the Animal Care and Use Committee of the Third Military Medical University, China (AMUWEC20230126).

### 5.2 Experimental Design

In the initial observational phase, we analyzed spatial exploration behaviors in both WT and AD mice to confirm disease‐related deficits. We also recorded and analyzed astrocytic Ca^2^^+^ transients associated with spatial exploration using fiber photometry. In the interventional phase, we assessed the therapeutic potential of GPC transplantation. Seven‐to‐eight‐month‐old AD mice received stereotaxic transplantation into the MECII. Three experimental groups were established: AD‐engrafted (GPC‐transplanted AD mice), AD‐control (sham‐operated AD mice), and WT‐control (sham‐operated WT mice). APP/PS1 mice were allocated to the AD‐control or AD‐engrafted group before surgery, whereas WT mice receiving sham surgery served as WT‐control mice. Histological, morphological, and behavioral analyses were conducted four months post‐transplantation.

### 5.3 Microinjection of Adeno‐Associated Virus (AAV)

To enable astrocyte‐specific expression of the GCaMP6f calcium indicator, we anesthetized mice with 1%–2% isoflurane and positioned them in a stereotaxic head frame on a heating pad (37.5°C–38°C) following standard protocols [36, 37]. After making a midline scalp incision, we performed a small craniotomy (0.5 × 0.5 mm) above the dorsal internal olfactory cortex.

We used an AAV vector encoding GCaMP6f under the control of the astrocyte‐specific GfaABC_1_D promoter (AAV5‐GfaABC_1_D‐cyto‐GCaMP6f‐SV40; Addgene #52925; titer ≥7 × 10^1^ ^2^ vg/mL). The viral suspension was injected into the MECII using a glass micropipette (10–20 µm tip diameter), which was connected to a 5 µL syringe (#75, Hamilton, United States) controlled by a syringe pump (788130, KD Scientific, United States). The injection was targeted to the following coordinates relative to Bregma: AP −5.0 mm and ML +3.2 mm. We delivered a total of 150 nL per hemisphere at a rate of 1 nL/s, along a dorsal‐ventral trajectory from −2.0 to −1.1 mm relative to the dural surface.

The micropipette was left in place for 10 min post‐injection to minimize backflow. Afterward, we closed the scalp using tissue adhesive (1469SB Vetbond, 3 M Animal Care Products, United States) and administered analgesics for 3 days following the surgery. All subsequent experiments were conducted 4–6 weeks post‐injection to allow for optimal viral expression.

### 5.4 Fiber‐Optic Photometric Recording of Freely Moving Mice

Four to six weeks post‐AAV injection, mice were prepared for fiber photometry according to established protocols [1, 2, 35, 109]. Under isoflurane anesthesia, we stereotaxically implanted an optic fiber cannula (907‐03007‐00, 1.25 mm OD, 200 µm core diameter, 0.39 numerical aperture, RWD Life Sciences, China) above the MECII injection site (AP −5.0 mm, ML +3.2 mm, DV −1.6 mm). The cannula was secured to the skull using dental cement.

Prior to recording, we habituated the mice for 3 consecutive days (5 min per day) to minimize stress and adapt to handling [1, 12, 109]. During recording sessions, we excited GCaMP6f fluorescence with a 470 nm LED and captured the emitted signal using a fiber photometry system (RWD). Simultaneously, animal behavior was video‐recorded at 30 Hz (1280 × 720 pixels) and synchronized with the photometric data stream.

### 5.5 Acquisition of Glial Progenitor Cells and Cell Transplantation

Neural stem cells (NSCs) were isolated from the cerebral cortices of E14.5‐15.5 ZsGreen1 reporter mouse embryos using mechanical dissociation, following a modified version of a previously published protocol [33]. The isolated cells were cultured as neurospheres in serum‐free NSC medium. To differentiate the neurospheres into GPCs, we dissociated them and cultured the cells for 2 days in medium containing 10 ng/mL ciliary neurotrophic factor (CNTF) (Sigma) and 30% fetal bovine serum (FBS) (Gibco), while omitting epidermal growth factor (EGF) and fibroblast growth factor 2 (FGF2). After differentiation, GPCs were dissociated using Accutase (eBioscience) and resuspended in phosphate‐buffered saline (PBS) at a final concentration of 4 × 10^5^ cells/µL for transplantation.

For transplantation, mice were anesthetized with isoflurane (1%–2%) and secured in a stereotaxic frame (68001, RWD Life Sciences, China). A microsyringe (65460‐02, Hamilton, United States) was positioned above the MECII at the following coordinates relative to Bregma: AP −5.0 mm, ML ±3.2 mm (bilateral). A total of 1.5 µL of GPC suspension or PBS vehicle was injected into each hemisphere, corresponding to 3.0 µL in total per mouse, at a rate of 5 nL/s. In each hemisphere, the injection was distributed across five vertical sites, with 300 nL deposited at 0.2 mm intervals starting at a depth of −2.0 mm from the dura. The syringe was held in place for 10 min post‐injection before being slowly withdrawn.

Intracisterna magna tracer injection: CSF tracer influx was assessed by intracisterna magna injection of fluorescent tracer as previously described with minor modifications [92, 95]. Mice were anesthetized with sodium pentobarbital and placed in the prone position in a stereotaxic frame on a heating pad maintained at 37°C. After shaving and disinfecting the posterior neck region, a midline skin incision was made, and the posterior cervical muscles were gently separated to expose the cisterna magna. OVA–Alexa Fluor 647 tracer solution was prepared at 5 mg mL^−^^1^, and 5 µL of tracer was loaded into a Hamilton syringe connected to PE‐10 tubing fitted with a 30‐gauge needle. The needle was advanced into the cisterna magna from the posterior side at an angle of approximately 45°, and tracer was infused at 1 µL min^−^^1^ using a microperfusion pump. After infusion, the needle was left in place for 5 min to minimize CSF reflux, slowly withdrawn, and the puncture site was sealed with tissue adhesive to prevent CSF leakage. Mice were transcardially perfused 30 min after tracer injection for fluorescence‐based analysis of tracer distribution.

### 5.6 Immunohistochemistry and Image Analysis

Mice were anesthetized with sodium pentobarbital (1 g/kg), and brain tissues were collected via cardiac perfusion with 4% paraformaldehyde (PFA) in PBS. The brain tissues were then dehydrated in a 15% sucrose PFA solution for 24 h and sectioned into 40 µm thick slices.

After 1 h of permeabilization and 2 h of blocking (with 10% normal donkey serum and 0.1% Triton X‐100 in PBS), brain sections were incubated overnight at 4°C with primary antibodies. The primary antibodies used included: rabbit anti‐S100β (287003, 1:500, SYSY), chicken anti‐GFP (ab13970, 1:500, Abcam), goat anti‐GFAP (ab53554, 1:500, Abcam), mouse anti‐6E10 (803015, 1:500, BioLegend), rabbit anti‐AQP4 (A5971, 1:500, Sigma), rat anti‐LAMP1 (121602, 1:500, BioLegend), rat anti‐CD31 (550274, 1:100, BD Biosciences), rabbit anti‐c‐Fos (226017, 1:400, Synaptic Systems), rabbit anti‐IBA1 (019‐19741, 1:200, Wako), rabbit anti‐D‐serine (IS1004, 1:300, ImmuSmol), rabbit anti‐CX43 (71‐0700, 1:300, Thermo Fisher), rabbit anti‐Synaptophysin (ab14692, 1:200, Abcam), mouse anti‐Nestin (ab22035, 1:200, Abcam), and mouse anti‐A2B5 (ab53521, 1:200, Abcam). Following primary antibody incubation, sections were rinsed in PBS and incubated for 2 h at room temperature in secondary antibodies (protected from light). The secondary antibodies used included: Alexa Fluor 488 donkey anti‐chicken (A78948, 1:800, Invitrogen), Alexa Fluor 594 donkey anti‐mouse (A21203, 1:800, Invitrogen), Alexa Fluor 647 donkey anti‐rabbit (A‐31573, 1:500, Invitrogen), Alexa Fluor 594 donkey anti‐rat (A21209, 1:800, Invitrogen), and Alexa Fluor 647 donkey anti‐goat (A21447, 1:800, Invitrogen). To label nuclei, brain sections were stained with 4′,6‐diamidino‐2‐phenylindole (DAPI).

Images were acquired using a Zeiss LSM 800 confocal microscope with ×20 (numerical aperture 0.50), ×40 (numerical aperture 1.30, oil), and ×63 (numerical aperture 1.40, oil) objectives. Surviving engrafted cells were quantified in three sections selected from 11 serial 40‐µm MECII sections and identified as ZsGreen1^+^ cells with clearly defined DAPI^+^ nuclei. The resolution was set to 1024 × 1024 pixels, and Z‐stacks were obtained where applicable, with a step size of 4 µm. For image analysis and quantification, we used Zeiss Efficient Navigation (ZEN) and Fiji (ImageJ) software [33, 110, 111].

For GCaMP6f/S100β co‐labeling analysis, GCaMP6f^+^ cells and S100β^+^ astrocytes within the MECII were manually counted from confocal images. Astrocytic labeling specificity was calculated as the percentage of GCaMP6f^+^ cells that were S100β^+^, and labeling efficiency was calculated as the percentage of S100β^+^ astrocytes that were GCaMP6f^+^ [37, 43].

For quantification of engrafted cell survival, ZsGreen1^+^ cells with clearly defined DAPI^+^ nuclei were counted in three sections selected from 11 serial 40‐µm MECII sections per mouse. The total number of surviving engrafted cells per mouse was estimated by scaling the counted cells according to the sampled fraction of MECII sections. Engraftment efficiency was calculated as the estimated number of surviving ZsGreen1^+^ cells divided by the total number of injected GPCs. For 0.5‐month graft maturation analysis, the percentage of GFAP^+^ZsGreen1^+^ cells among total ZsGreen1^+^ engrafted cells was quantified.

For perivascular AQP4 analysis, blood vessels were identified by CD31 immunolabeling or vessel‐like perivascular profiles. AQP4 fluorescence intensity was measured from line scans drawn perpendicular to the vessel wall, and AQP4 polarization was quantified as the difference between peak and baseline fluorescence intensity. In AD‐engrafted sections, graft‐associated perivascular regions were defined as perivascular regions adjacent to or ensheathed by ZsGreen1^+^ engrafted processes, whereas remote host‐only perivascular regions were defined as ZsGreen1‐negative host perivascular regions without adjacent visible engrafted processes in the analyzed field.

For c‐Fos analysis, c‐Fos^+^S100β^+^ cells were quantified among total S100β^+^ astrocytes. In AD‐engrafted mice, activity‐related labeling of engrafted cells was quantified as the percentage of c‐Fos^+^ZsGreen1^+^ cells among total ZsGreen1^+^ engrafted cells.

For tracer‐associated Aβ analysis, tracer‐positive perivascular regions were identified from OVA–Alexa Fluor 647 signal. Concentric distance bins were generated from tracer‐positive regions, and Aβ‐positive area fraction was calculated within each bin as Aβ‐positive pixels divided by the total area of the corresponding bin.

#### 5.6.1 Morphological Analysis

Astrocytes and microglia (Figures 3 and 5) were analyzed using Sholl analysis, total process length, and cellular territory measurements via built‐in plugins.

#### 5.6.2 Area Fraction and Intensity

For Aβ, LAMP1, and synaptophysin (SYP) (Figures 4, 5, and 6), images were thresholded to create binary masks, and area fraction (%) or mean pixel intensity was calculated.

#### 5.6.3 AQP4 Polarization

Perivascular AQP4 localization (Figure 4) was measured by fluorescence intensity from linescans drawn perpendicular to blood vessels.

#### 5.6.4 Co‐Localization Analysis

The spatial correlation between LAMP1 and Aβ (Figure S6) was quantified using the Pearson's correlation coefficient.

#### 5.6.5 Morphological Index

The morphological index was calculated as the ratio of soma area to arborization area [112, 113].

### 5.7 Behavioral Test

All mice were acclimated to the testing environment for at least 30 min prior to each experiment. The Open Field Test (OFT) was conducted before spatial exploration to assess voluntary locomotor activity and stress levels. Mice were placed in the center of a 50 cm × 50 cm × 50 cm open field box, with the central area 15 cm from the walls, facing away from the experimenter. An infrared camera mounted above the box recorded the mice's movements for 10 min. The resulting videos were processed using MATLAB software for motion tracking.

To evaluate spatial exploration behavior, we followed established methods from previous studies [12]. In these experiments, mice were placed in a 25 cm × 15 cm × 15 cm recording chamber. Each trial lasted 15 min, with the mice free to explore before being returned to their cage. Spatial exploration was quantified based on two specific behaviors: (1) “rearing,” where the mouse stood on its hind limbs, with its forelimbs touching the wall and its nose directed upward, and (2) “sniffing,” in which the mouse touched the wall with its nose [12]. Both behaviors were manually scored from the video recordings.

#### 5.7.1 Buried Food Test

Olfactory function was assessed using the buried food test, as described previously [114, 115]. To familiarize mice with the food reward and reduce neophobia, chocolate‐flavored food pellets were placed in the home cage for five consecutive days before testing. Mice were food‐deprived for 16 h before the test, with water available ad libitum. On the test day, mice were transferred to the behavioral room and habituated for 1 h. A clean test cage was filled with fresh bedding to a depth of approximately 3 cm. Each mouse was allowed to explore the test cage for 5 min and was then briefly removed. A single food pellet was buried 0.5–1 cm beneath the bedding surface at a randomly selected position. The mouse was then returned to the center of the test cage, and the latency to locate the buried pellet was recorded. Successful retrieval was defined as uncovering the pellet and beginning to eat it. Mice that failed to retrieve the pellet within 300 s were assigned a latency of 300 s. Bedding was replaced, and the test cage was cleaned between animals to eliminate residual olfactory cues.

#### 5.7.2 Y‐Maze Novel‐Arm Test

Spatial recognition memory was assessed using a novel‐arm Y‐maze paradigm as previously described [116, 117]. The apparatus consisted of three identical arms arranged at 120° angles. On day 1, mice were habituated to the maze by allowing free exploration for 10 min. On day 2, mice underwent a training phase, a 1 h retention interval, and a test phase. During training, one arm was blocked, and mice were allowed to freely explore the start arm and one familiar arm for 10 min. After the training session, mice were returned to their home cages for 1 h. During testing, the previously blocked arm was opened, and mice were allowed to freely explore all three arms for 10 min. Spatial recognition memory was quantified as the percentage of time spent in the novel arm.

#### 5.7.3 Novel Location Recognition Test

The novel location recognition test was performed in a 50 × 50 cm open‐field arena as previously described [118, 119]. On day 1, mice were placed in the empty arena and allowed to freely explore for 10 min. On day 2, mice underwent learning and testing phases. During learning, two identical objects were placed symmetrically in two distinct quadrants, and mice were allowed to explore for 10 min. After a 1 h retention interval, one object was relocated to a novel quadrant, whereas the other object remained in its original position. Mice were then reintroduced into the arena and allowed to explore for 10 min. The object location recognition index was calculated as the time spent exploring the object in the novel location divided by the total exploration time of both objects × 100%.

#### 5.7.4 Novel‐Environment Exposure for c‐Fos Analysis

To assess activity‐related recruitment of astrocytes and engrafted cells, mice were exposed to a novel open‐field environment for 15 min, whereas home‐cage control mice remained undisturbed in their home cages. Brain tissues were collected 105 min after session onset, corresponding to 90 min after novel‐environment exposure, to capture c‐Fos protein induction. This timing was selected based on the established temporal profile of c‐Fos protein expression after stimulation [120].

### 5.8 Data analysis and Statistics

We recorded Ca^2^^+^ transients in astrocytes of freely behaving mice using a fiber‐optic photometry technique at 470 nm. Ca^2^^+^ transient values were calculated using the formula ΔF/F = (*F*_raw_−*F*_baseline_) / F_baseline_ [121, 122]. The region of interest was selected from the fiber‐optic beam's grayscale image, where the average pixel intensity represented the raw Ca^2^^+^ signal (*F*_raw_). A moving window average of this signal served as the baseline (*F*_baseline_), and both were processed using OFRS software (RWD Life Sciences, China). To quantify the mice's movements relative to body size and analyze the relationship between astrocytic Ca^2^^+^ transients and spatial exploration, image frames were converted to binary format, highlighting the mice's shape based on image intensities. A Ca^2^^+^ transient was considered valid when its peak amplitude exceeded three standard deviations above the background noise. We focused analyses on Ca^2^^+^ transients recorded at the onset of exploratory behavior, using temporally shuffled data from the same session as a control. Data processing was performed using MATLAB 2017 (MathWorks) and Igor Pro 6.0.3.1 (WaveMetrics) [123].

Statistical analyses were performed using GraphPad Prism (version 8.0) and SPSS (version 25.0). Results are presented as Mean ± Standard Error of the Mean (SEM). Formal a priori power calculations were not performed for all cellular or image‐based quantifications. For quantifications analyzed with n = 15 observations per group, a sensitivity analysis indicated that a three‐group one‐way ANOVA design had 80% power at α = 0.05 to detect large effects of approximately Cohen's f = 0.48. For cellular and image‐based analyses, n refers to the number of quantified cells, sections, fields, or regions, and the number of contributing mice is specified in the corresponding figure legends. Investigators performing behavioral scoring, image quantification, and statistical analysis were blinded to group allocation. Unpaired comparisons between two groups were conducted using the independent sample Wilcoxon rank sum test [36, 37, 123]. For comparisons involving three or more groups, we used one‐way ANOVA followed by Bonferroni's post‐hoc test when variances were equal or Tamhane's T2 test when variances were unequal (Levene's test, *p* < 0.05). Differences were considered significant at *p* < 0.05, and non‐significant comparisons are indicated as “n.s.”.

## Author Contributions

K.Z., M.W., and F.W. designed the study. F.W., J.F., and X.Z. performed the main experiments. M.G. and C.C. completed the cell culture experiment. Z.Y., M.H., Z.C., S.Y., and T.T. conducted the immunohistochemistry experiments. F.W., J.F., X.Z., C.Y., J.L. and H.W. analyzed the data. K.Z., M.W., S.D. and X.C. wrote the manuscript with input from all coauthors.

## Conflicts of Interest

The authors declare no conflicts of interest.

## Supporting information




**Supporting File**: advs77139‐sup‐0001‐SuppMat.docx.

## Data Availability

The data that support the findings of this study are available from the corresponding author upon reasonable request.
