# Supplementary material for: Entorhinal Astrocyte Transplants Restore Spatial Exploration and Alleviate Amyloid‐Beta Pathology in Alzheimer's Mice
Source: Adv Sci (Weinh). 2026 Aug 24:e77139. Online ahead of print. doi: 10.1002/advs.77139 (PMC13502269; doi:10.1002/advs.77139)
Supplement: Supplementary file 1 [file ADVS-9999-e77139-s001.docx]

**Supplementary Fig. 1 *In vitro* generation and characterization of transplantable glial progenitor cells. A** Schematic of the experimental procedure. NSCs were isolated from ZsGreen1^+^ embryos, expanded as neurospheres, and differentiated into GPCs for subsequent transplantation and *in vivo* analysis. A parallel culture was further differentiated into astrocytes for *in vitro* characterization. **B** Undifferentiated NSCs forming ZsGreen1-positive neurospheres that consistently expressed the neural stem cell marker Nestin after 4 to 5 days in culture. **C** Cells displaying a bipolar morphology characteristic of GPCs after 2 days of culture in medium with CNTF and FBS. Cells were also immunopositive for the GPC marker A_2_B_5_. **D** Quantification of the GPC population, showing that 95.00% of cells were A2B5-positive following induction (n = 380 cells in 8 fields of view). **E** Cells acquiring a mature, stellate astrocyte morphology after 9 days of culture in medium with B27, FBS, and CNTF. Cells also expressed astrocyte-specific marker GFAP. **F** Quantification of the GFAP population, showing that 87.74% of cells were GFAP-positive following induction (n = 318 cells in 8 fields of view).

**Supplementary Fig. 2 Engrafted astrocytes migrate within the MEC of AD mice.** **A** Migration of engrafted astrocytes within the brain of AD mice. Red lines indicate migration distance of each transplanted astrocyte from the injection site. **B** Histograms showing distribution of migration distances of engrafted astrocytes from the injection site in MEC (green bars). Black curve represents the fitted migration distribution (n = 110 cells).

**Supplementary Fig. 3 Engrafted ZsGreen1⁺ astrocytes ensheath host CD31⁺ blood vessels and form AQP4-positive perivascular endfoot-like structures within the MECII.** **A** Representative confocal images showing the labeling of CD31, AQP4, and ZsGreen1 in the MECII of AD-engrafted mice. The boxed region in the left image is presented at a higher magnification on the right. White dashed lines delineate the host CD31⁺ blood vessel. ZsGreen1⁺ engrafted astrocytic processes and AQP4 immunoreactivity are observed encircling the CD31⁺ vessel. **B** Representative confocal images showing a cross-sectional view of a CD31⁺ host blood vessel within the MECII of AD-engrafted mice. The boxed region in the left image is presented at higher magnification on the right. White dashed lines delineate host CD31⁺ vessels. ZsGreen1⁺ engrafted astrocytic processes encircle these vessels, forming AQP4-positive perivascular endfoot-like structures.

**Supplementary Fig. 4 Perivascular AQP4 intensity in MECII of AD-engrafted mice: regions with vs. without engrafted astrocytic processes. A** Average AQP4 fluorescence intensity profiles were analyzed across perivascular regions in the WT-control, AD-control, AD-host, and AD-engrafted groups. The WT-control regions (WT-contr.) and AD-control regions (AD-contr.) are defined as perivascular domains in the MECII of WT-control and AD-control mice, respectively. The AD-host regions (AD-host) are defined as perivascular domains that lack engrafted astrocyte processes, while the AD-engrafted regions (AD-engr.) are characterized by the presence of engrafted astrocyte processes. Line scans were aligned to the peak AQP4 fluorescence in the perivascular regions (n = 30 vessels per group from 6 mice). **B** Quantification of perivascular AQP4 intensity was performed by calculating the difference between peak and baseline fluorescence intensity. The data for WT-control and AD-control have been re-plotted from Fig. 4E for reference. Measurements for AD-host and AD-engr. were quantified from brain slices obtained from AD-engrafted mice (n = 30 vessels per group from 6 mice). **P* < 0.05, ***P* < 0.01, ****P* < 0.001, via one-way ANOVA with Tamhane's T2 post hoc comparisons test. All data are presented as mean ± SEM.

**Supplementary Fig. 5 Glymphatic CSF tracer distribution connects the expression of AQP4 to the distance-dependent accumulation of Aβ in the MECII.**

**A** Schematic illustration of intracisterna magna (i.c.m.) injection of a fluorescent CSF tracer. The tracer was delivered into the cisterna magna and distributed through cisterna magna–connected CSF spaces (grey). **B** Representative confocal image from the MECII showing intracisternally delivered CSF tracer along a CD31-positive vascular profile and in close apposition to AQP4-positive perivascular astrocytic endfeet. **C** Representative image showing concentric radial distance bins centered on a tracer-positive region for quantifying the percentage of Aβ-positive area in each bin. **D** Quantification of Aβ-positive area as a percentage of the total area within each distance bin from tracer-positive regions (n = 15 tracer-associated regions from 5 mice; each region contributed one value to each distance bin). Data are presented as mean ± SEM. ****P* < 0.001, via one-way ANOVA with Tamhane's T2 post hoc comparisons test.

**Supplementary Fig. 6 Spatial association between LAMP1 and Aβ plaques in AD-control and AD-engrafted mice. A** Representative images of LAMP1 (pink) and Aβ plaque (cyan) immunofluorescence colocalization in AD-control mice (top) and AD-engrafted mice (bottom). **B** Histograms summarizing Pearson correlation coefficients of LAMP1 and Aβ immunofluorescence colocalization in AD-control and AD-engrafted mice (n = 15 brain slices per group from 5 mice). **C** Histograms summarizing quantification of percentage of Aβ plaques that are immunopositive for LAMP1 in AD-control and AD-engrafted mice (n = 15 brain slices per group from 5 mice). Independent samples via the Wilcoxon rank sum test. All data are presented as mean ± SEM.

**Supplementary Fig. 7 Buried food test for assessment of olfactory function in WT-control, AD-control, and AD-engrafted mice. A** Schematic illustration of the buried food test procedure. Mice were habituated to the food reward for 5 days, followed by 16 h of food deprivation with water available ad libitum. On the test day, mice were habituated to the behavioral room for 1 h and then allowed to explore the test cage for 5 min. The latency to locate the buried food pellet was recorded, with a maximum cutoff time of 300 s. **B** Quantification of the latency to locate the buried food pellet in WT-control, AD-control and AD-engrafted mice (n = 8 mice per group), **P* < 0.05, ***P* < 0.01, ****P* < 0.001, via one-way ANOVA with Tamhane's T2 post hoc comparisons test. Data are presented as mean ± SEM.

**Supplementary Fig. 8 GFAP co-labeling and spatial exploration assessment in AD-engrafted mice at 0.5 months post-transplantation. A** Representative confocal image showing GFAP and ZsGreen1 labeling in the MECII of AD-engr-0.5 M mice. **B** Quantification of the percentage of GFAP⁺ZsGreen1⁺ cells among total ZsGreen1⁺ engrafted cells in AD-engr-0.5 M mice (n = 15 brain slices from 5 mice). Data are presented as mean ± SEM. **C-E** Quantification of spatial exploration frequency **(C)**, single-trial spatial exploration duration **(D)**, and total spatial exploration time **(E)** in WT-control, AD-control, AD-engrafted mice at 0.5 months post-transplantation (AD-engr-0.5 M), and AD-engrafted mice at 4 months post-transplantation (AD-engr-4 M). WT-control, AD-control, and AD-engr-4 M behavioral data are re-plotted from Fig. 7 for comparison with the newly added AD-engr-0.5 M cohort (n = 10 mice per group), **P* < 0.05, ***P* < 0.01, ****P* < 0.001, via one-way ANOVA with Bonferroni post hoc comparisons test. All data are presented as mean ± SEM.

**Supplementary Fig. 9 c-Fos labeling in engrafted cells following** **home-cage (HC) exposure or novel-environment (NE) spatial exploration.** A Experimental design for HC and NE groups. HC mice remained in their home cages, whereas NE mice explored a novel chamber for 15 min; brains were collected at 105 min for post hoc immunohistochemistry. **B** Quantification of c-Fos⁺S100β⁺ double-positive cells relative to total S100β⁺ cells in WT-HC, WT-NE, AD-engr-HC and AD-engr-NE groups (n = 15 brain slices per group, 5 mice per experimental group). ****P* < 0.001, via one-way ANOVA with Tamhane's T2 post hoc comparisons test. **C** Representative confocal images showing c-Fos and ZsGreen1 labeling in the MECII of AD-engrafted mice after NE exploration. The boxed region is enlarged on the right. Arrowheads indicate c-Fos⁺ZsGreen1⁺ engrafted cells. **D** Quantification of c-Fos⁺ZsGreen1⁺ double-positive cells relative to total ZsGreen1⁺ engrafted cells in AD-engr-HC and AD-engr-NE groups (n = 15 brain slices per group, 5 mice per experimental group). ****P* < 0.001, independent samples via the Wilcoxon rank sum test. All data are presented as mean ± SEM.

**Supplementary Fig. 10 Spatial memory assessment after MECII astrocyte transplantation in AD mice. A** Schematic of the Y-maze novel-arm test. During the training session, one arm was closed for 10 minutes. One hour later, the mice were tested for 10 minutes with all three arms open. **B** Representative occupancy heatmaps of WT-control, AD-control, and AD-engrafted mice during the Y-maze test. **C** Quantification of the percentage of time spent in the novel arm (n = 8 mice per group), **P* < 0.05, ***P* < 0.01, ****P* < 0.001, via one-way ANOVA with Bonferroni post hoc comparisons test. **D** Schematic of the novel location recognition test. During the training session, mice were allowed to explore two identical objects for a duration of 10 minutes. One hour later, one of the objects was relocated to a novel position, and the mice were then tested for an additional 10 minutes. **E** Representative occupancy heatmaps of WT-control, AD-control, and AD-engrafted mice during the novel location recognition test. **F** Quantification of the percentage of object exploration time spent at the novel location (n = 8 mice per group). **P* < 0.05, ***P* < 0.01, ****P* < 0.001, via one-way ANOVA with Bonferroni post hoc comparisons test. Data are presented as mean ± SEM.
